# The effect of 90-90-90 on HIV-1 incidence and mortality in eSwatini: a mathematical modelling study

**DOI:** 10.1016/S2352-3018(19)30436-9

**Published:** 2020-02-13

**Authors:** Adam Akullian, Michelle Morrison, Geoffrey P Garnett, Zandile Mnisi, Nomthandazo Lukhele, Daniel Bridenbecker, Anna Bershteyn

**Affiliations:** a Institute for Disease Modeling, Bellevue, WA, USA; b Department of Global Health, University of Washington, Seattle, WA, USA; c Bill & Melinda Gates Foundation, Seattle, WA, USA; d Ministry of Health, Kingdom of eSwatini, Mbabane, eSwatini; e World Health Organization, eSwatini Country Office, Mbabane, eSwatini; f Department of Population Health, New York University School of Medicine, New York, NY, USA

## Abstract

**Background:**

The rapid scale-up of antiretroviral therapy (ART) towards the UNAIDS 90-90-90 goals over the last decade has sparked considerable debate as to whether universal test and treat can end the HIV-1 epidemic in sub-Saharan Africa. We aimed to develop a network transmission model, calibrated to capture age-specific and sex-specific gaps in the scale-up of ART, to estimate the historical and future effect of attaining and surpassing the UNAIDS 90-90-90 treatment targets on HIV-1 incidence and mortality, and to assess whether these interventions will be enough to achieve epidemic control (incidence of 1 infection per 1000 person-years) by 2030.

**Methods:**

We used eSwatini (formerly Swaziland) as a case study to develop our model. We used data on HIV prevalence by 5-year age bins, sex, and year from the 2007 Swaziland Demographic Health Survey (SDHS), the 2011 Swaziland HIV Incidence Measurement Survey, and the 2016 Swaziland Population Health Impact Assessment (PHIA) survey. We estimated the point prevalence of ART coverage among all HIV-infected individuals by age, sex, and year. Age-specific data on the prevalence of male circumcision from the SDHS and PHIA surveys were used as model inputs for traditional male circumcision and scale-up of voluntary medical male circumcision (VMMC). We calibrated our model using publicly available data on demographics; HIV prevalence by 5-year age bins, sex, and year; and ART coverage by age, sex, and year. We modelled the effects of five scenarios (historical scale-up of ART and VMMC [status quo], no ART or VMMC, no ART, age-targeted 90-90-90, and 100% ART initiation) to quantify the contribution of ART scale-up to declines in HIV incidence and mortality in individuals aged 15–49 by 2016, 2030, and 2050.

**Findings:**

Between 2010 and 2016, status-quo ART scale-up among adults (aged 15–49 years) in eSwatini (from 34·0% in 2010 to 74·1% in 2016) reduced HIV incidence by 43·57% (95% credible interval 39·71 to 46·36) and HIV mortality by 56·17% (54·06 to 58·92) among individuals aged 15–49 years, with larger reductions in incidence among men and mortality among women. Holding 2016 ART coverage levels by age and sex into the future, by 2030 adult HIV incidence would fall to 1·09 (0·87 to 1·29) per 100 person-years, 1·42 (1·13 to 1·71) per 100 person-years among women and 0·79 (0·63 to 0·94) per 100 person-years among men. Achieving the 90-90-90 targets evenly by age and sex would further reduce incidence beyond status-quo ART, primarily among individuals aged 15–24 years (an additional 17·37% [7·33 to 26·12] reduction between 2016 and 2030), with only modest additional incidence reductions in adults aged 35–49 years (1·99% [–5·09 to 7·74]). Achieving 100% ART initiation among all people living with HIV within an average of 6 months from infection—an upper bound of plausible treatment effect—would reduce adult HIV incidence to 0·73 infections (0·55 to 0·92) per 100 person-years by 2030 and 0·46 (0·33 to 0·59) per 100 person-years by 2050.

**Interpretation:**

Scale-up of ART over the last decade has already contributed to substantial reductions in HIV-1 incidence and mortality in eSwatini. Focused ART targeting would further reduce incidence, especially in younger individuals, but even the most aggressive treatment campaigns would be insufficient to end the epidemic in high-burden settings without a renewed focus on expanding preventive measures.

**Funding:**

Global Good Fund and the Bill & Melinda Gates Foundation.

## Introduction

In 2014, UNAIDS, as part of new universal test and treat guidelines, put forth the 90-90-90 goals,^1^ galvanising high-burden countries in sub-Saharan Africa to attain 73% viral load suppression among people living with HIV by 2020, with the goal of achieving epidemic control (defined as annual incidence of <1 infection per 1000 individuals, or <0·1 infections per 100 person-years) by 2030.^2^ The 90-90-90 goals and universal test and treat in general have been widely adopted across sub-Saharan Africa, and the implementation of rapid scale-up of antiretroviral therapy (ART) has resulted in millions of lives saved.^3^

Whether the continued expansion of treatment targets will be enough to achieve epidemic control remains unclear, given large uncertainty around the effectiveness of universal test and treat in real-world settings.^4^ Universal test and treat might only suffice to stabilise or modestly reduce incidence over time in the most intense epidemics. Substantial reductions in new HIV infections have followed expanded ART coverage in some regions of east and southern Africa,^5^, ^6^ although incidence after ART scale-up has been well above predictions from previous models.^7^ Incidence reductions have been more modest in other regions, including in the hyperepidemic province of KwaZulu-Natal (South Africa),^8^ where incidence has not declined as much as expected given the expansion of treatment eligibility.^9^

**Research in context**

**Evidence before this study**

We searched PubMed from Jan 1, 2004, to April 1, 2019, with the terms “HIV”[Title] AND (“modeling”[Title] OR “model”[Title] OR “modelling”[Title])) AND “Africa”[All Fields]) AND (“Treatment as prevention”[Title] OR “Test and treat”[Title] OR “Antiretroviral therapy”[Title] OR “ART”[Title]). There is clear evidence to support early treatment of people living with HIV to reduce population-level incidence. Multiple mathematical models have projected that epidemic control of HIV is theoretically possible with the adoption of universal test and treat. As multiple countries in sub-Saharan Africa begin to achieve ambitious treatment targets, including those specified by the UNAIDS 90-90-90 goals, attention has turned to understanding how demographic gaps in antiretrovira therapy (ART) coverage, especially suboptimal coverage in younger men and women living with HIV, attenuate the effects of ART scale-up on HIV incidence reductions. No mathematical model to date has evaluated the effects of treatment gaps on the future of the epidemic and whether closing those gaps will result in epidemic control.

**Added value of this study**

The scale-up of ART in eSwatini since 2004 has dramatically reduced HIV incidence and mortality and is a valuable case study to investigate whether the achievement of high ART coverage will be enough to move the country towards epidemic control. Our study projects the effect of the current ART programme on HIV incidence and mortality with a specific focus on capturing differential ART coverage by age and sex. Considering realistic assumptions of the HIV care cascade, we found that reaching and even surpassing the 90-90-90 targets will not be enough to end the epidemic, given the attenuating effects of suboptimal treatment coverage in the demographic groups that sustain HIV transmission, imperfect adherence to ART, and acute and early transmission. Even under the most aggressive ART intervention, in which 100% of all infected individuals initiate treatment soon after infection, HIV incidence is still expected to stay above the epidemic control target of 1 infection per 1000 person-years.

**Implications of all the available evidence**

Although ART remains one of the most effective interventions to control HIV transmission, achieving epidemic control and eventual elimination in high-burden settings like eSwatini will require an increased emphasis on expanding prevention to the highest risk groups, including new tools and implementation strategies. Expanding treatment targets will continue to push down incidence but not to epidemic control levels.

Part of the reason that universal test and treat has not produced the dramatic incidence reductions predicted in previous mathematical models is that although treatment eligibility is universal, coverage is not. Large demographic gaps in HIV testing, ART uptake, and viral load suppression are pervasive in the era of universal test and treat;^10^ even state-of-the-art test and treat interventions have faced challenges in reaching the 90-90-90 targets in younger age groups, especially in young men.^11^ Universal test and treat often misses demographic groups who disproportionately transmit HIV in high-burden settings,^12^ threatening the overall population-level effectiveness of treatment targets. The effect of heterogeneity in ART coverage on population-level incidence patterns is potentially substantial but has not been adequately quantified nor incorporated into dynamic modelling.^13^

eSwatini (formerly Swaziland) has the world's highest HIV prevalence and is close to achieving the UNAIDS 90-90-90 targets of viral load suppression in 73% of people living with HIV, although suppression was only 33% among young men in 2016.^14^ The recent scale-up of ART in eSwatini, which doubled the prevalence of viral load suppression, is cited as the primary driver of the steep decline in adult HIV incidence from 2010 to 2016.^15^ Given demographic gaps in treatment coverage, however, the future effect of achieving treatment targets is unclear, and there is need for a rigorous evaluation of whether the expansion of universal test and treat in countries such as eSwatini will be enough to achieve epidemic control considering demographic heterogeneity across the care cascade.^16^ Additionally, ART has scaled up in eSwatini alongside other prevention modalities, including voluntary medical male circumcision (VMMC). The relative contribution to incidence reduction from each modality has not been rigorously assessed.

We used an individual-based, network transmission model of HIV-1, explicitly calibrated to the age-specific and sex-specific scale-up of ART and VMMC, to simulate historical and future incidence and mortality trajectories in eSwatini. The model is used to quantify the contribution of ART to historical declines in HIV incidence and mortality in adults (herein defined as individuals aged 15–49 years) in eSwatini, provide insight into whether attaining current treatment targets will be enough to achieve epidemic control by 2030, explore potential expansion of the 90-90-90 targets by subgroup, and establish a theoretical upper bound on HIV incidence reductions driven by universal test and treat in the world's highest prevalence nation.

## Methods

### Model description

Microsimulation modelling was implemented using the Epidemiological MODelling software (EMOD), which is described in detail in Institute of Disease Modeling (Bellevue, WA, USA) documentation and elsewhere.^17^ EMOD is an open-source, stochastic, agent-based model that simulates within-host disease progression and between-host sexual interactions of individuals to understand the dynamics of disease transmission within a specific epidemic and health-care context. EMOD incorporates data on age-specific fertility, mortality, and sexual relationship formation and explicitly models the flow of individuals through a configurable HIV cascade of care—including testing, time-variable linkage to care, retention in care, treatment eligibility, and retention on ART.

EMOD uses three key structural components to simulate the age-specific, sex-specific, and risk-group-specific sexual network and the age-specific and sex-specific scale-up of ART. The first is a sexual partner pair formation algorithm,^18^ which simulates a detailed representation of the age-specific structuring of heterosexual networks—a phenomenon that contributes to the age-specific and sex-specific patterns of HIV transmission.^19^ Using data on validated, self-reported patterns of age-specific sexual partners,^20^ EMOD recapitulates age-specific transmission dynamics,^18^ as confirmed by epidemiological and phylogenetic studies in nearby KwaZulu-Natal, South Africa.^19^, ^21^

The second key component in EMOD is the natural history of HIV progression and transmission in the presence or absence of treatment. The health effect of ART must consider variable health states (such as CD4 cell count and WHO disease stage), at the time of ART initiation and over cycles of ART interruption and re-initiation. Lastly, any model of ART effect must consider the HIV continuum of care, including differential access to testing, linkage, retention, and viral suppression for subpopulations such as pregnant women, children, and individuals experiencing AIDS-related symptoms. This component enables EMOD to depict ART scale-up through different configurations of testing and service provision and provides a more realistic representation of the immediate health effect of ART on reducing AIDS-related death, as opposed to the longer-term effect of early ART initiation on reducing onward transmission, than do other HIV models. ART is assumed to reduce infectiousness on average by 92%,^22^ an estimate that incorporates real-world barriers to suppression, including adherence and undetected drug resistance. The transmission benefit of ART is assumed to grow linearly over the first 6 months after ART initiation.

EMOD is further configured to capture population heterogeneity by risk group (based on extra-partnership parameters) and relationship type (“marital”, which is long in duration and more likely to involve older cohabiting individuals; “informal”, which are intermediate in duration, non-marital relationships with individuals of marital age or younger; “transitory”, which is short term and involving younger individuals; and “commercial”, which is short in duration and has high turnover and concurrency) and can target interventions at specific groups defined by these individual-specific and partner-specific properties. This configurability allows the user to test the effects of differential uptake and access to treatment and prevention by specific demographic and risk strata. We assumed no changes in sexual risk behaviour following uptake of VMMC or ART in our model.

We modelled a closed population (no in or out migration), given the similar HIV prevalence in eSwatini to that of surrounding provinces of South Africa, a common destination for labour migrants. We furthermore assumed a country-level geography given that HIV prevalence is relatively homogeneous across the four provinces of eSwatini (varying from 25·7% to 29·4%).^14^

### Data sources

We used data on HIV prevalence by 5-year age bins, sex, and year from three population-based, country-wide surveys: the 2007 Swaziland Demographic Health Survey (SDHS),^23^ the 2011 Swaziland HIV Incidence Measurement Survey (SHIMS) survey,^24^, ^25^ and the 2016 Swaziland Population Health Impact Assessment (PHIA; also known as SHIMS2) survey^15^ (appendix pp 1, 2). We estimated the point prevalence of ART coverage among all HIV-infected individuals by age, sex, and year using data on viral load suppression (<1000 copies per mL) from the 2011 SHIMS^25^ and the 2016 PHIA.^15^ Because ART coverage data were not reported directly, we back calculated ART coverage from the prevalence of viral load suppression among HIV-infected individuals in eSwatini by sex and age group (15–24 years, 25–34 years, and 35–44 years),^14^, ^15^, ^22^ assuming that 90% of individuals on ART are virally suppressed. ART coverage for non-survey years (2005–10 and 2012–15) was imputed proportionally to annual relative changes in the crude number of individuals on ART over time.^26^

Age-specific data (in 5-year age groups from 15 years to 59 years) on the prevalence of male circumcision from two population-based surveys were used as model inputs for traditional male circumcision (2007 SDHS survey^23^) and scale-up of VMMC (2016 PHIA survey^15^). The prevalence of male circumcision between the two surveys was interpolated linearly and was held at 2016 levels for all modelling scenarios projecting into the future.

### Model calibration

We calibrated our model using publicly available data on demographics (fertility by age, mortality by age and sex, and the age distribution of the population); HIV prevalence by 5-year age bins, sex, and year; and ART coverage by age, sex, and year. Model calibration was done using a parallel simultaneous perturbation optimisation (PSPO) algorithm,^27^ which maximises the pseudolikelihood of stochastic epidemiological models given observed data and identifies an optimal set of input parameters. PSPO was run until the likelihood reached a plateau without further improvement. 250 model parameter sets were selected from 23 100 simulations using roulette resampling in proportion to the likelihood. The model calibration process produced a set of 25 fitted parameters whose distributions are described along with static parameters in the appendix (pp 3–9).

### Modelling scenarios

We estimated the contribution of treatment scale-up to reductions in HIV incidence and mortality by simulating an alternative history of the HIV epidemic in eSwatini in the absence of ART. We modelled the effects of five scenarios (table 1) on incidence and mortality among individuals aged 15–49 years. Methods used to estimate modelled effect estimates (prevalence, incidence, cumulative incidence reduction, and cumulative mortality reduction) are described in the appendix (p 11). We also estimated the proportion of transmissions originating from individuals in the acute and early phases of infection, the proportion of transmissions originating from individuals according to ART status (never initiated ART, on ART, and dropped out from ART), and the proportion of new acquisitions in the 15–24-years age group, under different scenarios. Sensitivity analyses were done to evaluate the contribution of uncertainty from four key parameters (acute-stage duration, acute-stage infectivity multiplier, time from infection to ART initiation, and ART efficacy) to incidence estimates for scenario 1 in our model (appendix p 16). We also did a sensitivity analysis of ART efficacy and time from infection to ART initiation for scenario 5 (100% ART initiation; appendix p 21).

**Table 1:** Modelling scenarios

	**Name**	**Description**
Scenario 1	Historical scale-up of ART and VMMC (status quo)	Status-quo scenario to match the historical scale-up of ART and VMMC by age and sex, maintaining 2016 levels of both interventions into the future
Scenario 2	No ART, no VMMC	Counterfactual scenario without ART or VMMC
Scenario 3	No ART (VMMC only)	Counterfactual scenario without ART (keeping historical VMMC scale-up and holding 2016 VMMC coverage levels); this scenario is compared with scenario 2 (no ART no VMMC) to quantify the effects of VMMC on incidence and mortality
Scenario 4	Age-targeted 90-90-90	Future scenario in which the 90-90-90 goals (81% ART coverage) are reached by broad age and sex groups (male and female; 15–24 years, 25–34 years, 35–44 years, and ≥45 years) by 2020 and held thereafter. Groups with greater than 81% ART coverage in the status-quo scenario were held at that coverage level
Scenario 5	100% ART initiation	Future scenario in which 100% of people living with HIV/AIDS initiate ART, on average, 6 months after seroconversion and achieve the full transmission benefit of ART 1 year after infection; cross-sectional coverage at any timepoint will be less than 100% because of the lag in ART uptake

ART=antiretroviral therapy. VMMC=voluntary medical male circumcision.

All statistical analyses and plotting were done in R version 3.6.1.

### Role of the funding source

The funder of the study played no role in study design, data collection, data analysis, data interpretation, or writing of the report. The corresponding author had full access to all the data in the study and had final responsibility for the decision to submit for publication.

## Results

**Figure 1:**
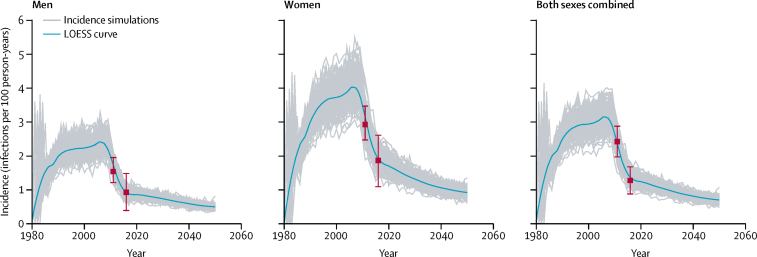
Best fitting incidence simulations (n=250) and LOESS curves for individuals aged 15–49 years by sex Data points are incidence point estimates (95% CI) from two population-based surveys (Swaziland HIV Incidence Measurement Survey, 2011,^24^ and Swaziland Population Health Impact Assessment, 2016).^15^ LOESS=locally weighted smoothing.

We modelled HIV incidence from 1980 to 2050 among men, women, and individuals of both sexes combined aged 15–49 years across 250 parameter sets (figure 1). After initial epidemic seeding in the early 1980s, adult incidence rose steadily to a peak of more than 3 infections per 100 person-years by 2006, then declined precipitously from 2010 to 2016, concurrent with ART roll-out. Incidence continued to decline after 2016, although at a slower rate because of holding ART at 2016 levels into the future. By 2016, according to scenario 1, annual incidence in men (0·94 infections, 95% credible interval [CrI] 0·72–1·16, per 100 person-years) was half that of women (2·01 infections, 1·54–2·49, per 100 person-years; table 2). Fitted model output, including prevalence over time by age and sex are shown in the appendix (p 10).

**Table 2:** Effect of intervention scenarios on the HIV epidemic outcomes in eSwatini over different time horizons (2010–16, 2016–30, and 2016–50) for adults aged 15–49 years, overall and by sex

		**Cumulative incidence reduction**	**Cumulative mortality reduction**	**Year**	**Incidence (per 100 person-years)**	**Proportion of new cases in 15–24 age group**
**Scenario 1: historical scale-up of ART and VMMC, maintaining 2016 levels (measures relative to scenario 3)**						
All						
	2010–16	43·57% (39·71 to 46·36)	56·17% (54·06 to 58·92)	2016	1·42 (1·12 to 1·71)	0·39 (0·34 to 0·44)
	2016–30	64·81% (61·69 to 67·14)	89·18% (88·38 to 89·88)	2030	1·09 (0·87 to 1·29)	0·26 (0·22 to 0·30)
	2016–50	70·27% (67·45 to 72·56)	93·00% (92·37 to 93·52)	2050	0·74 (0·60 to 0·91)	0·24 (0·19 to 0·29)
Females						
	2010–16	41·11% (37·44 to 44·59)	61·22% (58·76 to 63·45)	2016	2·01 (1·54 to 2·49)	0·48 (0·40 to 0·53)
	2016–30	64·18% (61·16 to 66·88)	92·99% (92·28 to 93·65)	2030	1·42 (1·13 to 1·71)	0·35 (0·28 to 0·40)
	2016–50	70·77% (67·79 to 73·13)	95·55% (95·17 to 95·93)	2050	0·97 (0·78 to 1·20)	0·30 (0·23 to 0·35)
Males						
	2010–16	47·02% (42·41 to 50·20)	48·94% (45·92 to 52·91)	2016	0·94 (0·72 to 1·16)	0·25 (0·18 to 0·34)
	2016–30	66·97% (63·66 to 68·95)	83·12% (81·54 to 84·44)	2030	0·79 (0·63 to 0·94)	0·14 (0·08 to 0·19)
	2016–50	71·72% (68·33 to 73·86)	88·68% (87·55 to 89·64)	2050	0·51 (0·40 to 0·64)	0·13 (0·08 to 0·18)
**Scenario 2: no ART, no VMMC**						
All						
	2010–16	..	..	2016	3·84 (3·03 to 4·65)	0·36 (0·32 to 0·40)
	2016–30	..	..	2030	4·24 (3·40 to 4·95)	0·34 (0·29 to 0·38)
	2016–50	..	..	2050	4·46 (3·64 to 5·23)	0·30 (0·26 to 0·35)
Females						
	2010–16	..	..	2016	4·89 (3·91 to 6·11)	0·45 (0·40 to 0·50)
	2016–30	..	..	2030	5·42 (4·29 to 6·42)	0·44 (0·38 to 0·50)
	2016–50	..	..	2050	5·81 (4·47 to 7·01)	0·41 (0·36 to 0·47)
Males						
	2010–16	..	..	2016	2·96 (2·39 to 3·55)	0·24 (0·19 to 0·29)
	2016–30	..	..	2030	3·30 (2·66 to 3·92)	0·21 (0·17 to 0·26)
	2016–50	..	..	2050	3·55 (2·89 to 4·30)	0·17 (0·13 to 0·20)
**Scenario 3: no ART (VMMC only; measures relative to scenario 2)**						
All						
	2010–16	4·56% (1·24 to 8·56)	0·60% (−2·14 to 3·52)	2016	3·60 (2·88 to 4·27)	0·35 (0·31 to 0·39)
	2016–30	12·26% (9·97 to 16·60)	4·53% (2·15 to 6·73)	2030	3·41 (2·82 to 3·95)	0·30 (0·25 to 0·33)
	2016–50	20·57% (18·51 to 23·90)	12·29% (10·44 to 15·23)	2050	3·15 (2·56 to 3·65)	0·25 (0·22 to 0·29)
Females						
	2010–16	3·03% (−0·52 to 6·73)	0·36% (−2·91 to 3·72)	2016	4·66 (3·74 to 5·74)	0·44 (0·39 to 0·49)
	2016–30	9·75% (6·61 to 13·49)	2·86% (−0·02 to 5·70)	2030	4·54 (3·69 to 5·46)	0·39 (0·34 to 0·45)
	2016–50	17·08% (14·65 to 20·79)	8·90% (6·92 to 11·87)	2050	4·27 (3·36 to 5·02)	0·35 (0·30 to 0·40)
Males						
	2010–16	7·17% (2·78 to 12·12)	1·06% (−2·33 to 4·91)	2016	2·67 (2·22 to 3·22)	0·22 (0·17 to 0·28)
	2016–30	15·74% (13·26 to 20·43)	6·92% (3·99 to 9·77)	2030	2·51 (2·10 to 3·03)	0·16 (0·12 to 0·20)
	2016–50	24·64% (22·58 to 27·68)	16·87% (14·88 to 20·19)	2050	2·36 (1·89 to 2·74)	0·13 (0·10 to 0·16)
**Scenario 4: ART scaled-up to 90-90-90 within each age and sex group (measures relative to scenario 3)**						
All						
	2010–16	43·32% (39·49 to 46·46)	56·76% (53·53 to 59·51)	2016	1·38 (1·14 to 1·72)	0·39 (0·33 to 0·45)
	2016–30	68·54% (65·61 to 71·01)	91·10% (90·30 to 91·84)	2030	0·97 (0·76 to 1·15)	0·23 (0·18 to 0·28)
	2016–50	73·66% (70·63 to 75·86)	94·61% (94·05 to 95·09)	2050	0·64 (0·51 to 0·81)	0·21 (0·16 to 0·26)
Females						
	2010–16	40·60% (36·47 to 44·63)	61·63% (58·94 to 64·13)	2016	1·97 (1·56 to 2·52)	0·47 (0·40 to 0·54)
	2016–30	68·20% (65·29 to 71·40)	93·74% (93·02 to 94·27)	2030	1·25 (0·97 to 1·52)	0·30 (0·24 to 0·36)
	2016–50	74·22% (71·25 to 76·65)	96·20% (95·79 to 96·57)	2050	0·85 (0·66 to 1·07)	0·26 (0·19 to 0·32)
Males						
	2010–16	46·89% (42·16 to 49·96)	49·47% (44·66 to 53·13)	2016	0·91 (0·73 to 1·14)	0·25 (0·18 to 0·32)
	2016–30	69·74% (66·36 to 72·20)	86·98% (85·81 to 88·09)	2030	0·71 (0·56 to 0·84)	0·12 (0·07 to 0·17)
	2016–50	74·63% (70·94 to 76·68)	91·98% (91·20 to 92·73)	2050	0·45 (0·31 to 0·58)	0·12 (0·06 to 0·18)
**Scenario 5: 100% ART initiation among all people living with HIV/AIDS within 6 months, on average, of infection (measures relative to scenario 3)**						
All						
	2010–16	43·48% (40·17 to 47·04)	56·10% (54·11 to 58·76)	2016	1·39 (1·07 to 1·62)	0·40 (0·34 to 0·46)
	2016–30	74·95% (72·23 to 76·84)	94·52% (94·05 to 94·93)	2030	0·73 (0·55 to 0·92)	0·21 (0·16 to 0·28)
	2016–50	80·13% (77·33 to 81·92)	97·39% (97·21 to 97·63)	2050	0·46 (0·33 to 0·59)	0·20 (0·12 to 0·24)
Females						
	2010–16	41·36% (37·53 to 44·85)	61·12% (58·67 to 63·25)	2016	1·97 (1·45 to 2·37)	0·48 (0·41 to 0·53)
	2016–30	74·83% (72·39 to 77·01)	95·91% (95·55 to 96·35)	2030	0·95 (0·68 to 1·18)	0·28 (0·20 to 0·36)
	2016–50	80·48% (77·60 to 82·57)	98·02% (97·83 to 98·22)	2050	0·60 (0·43 to 0·76)	0·24 (0·14 to 0·30)
Males						
	2010–16	47·18% (43·27 to 51·38)	48·67% (45·89 to 52·58)	2016	0·88 (0·67 to 1·10)	0·26 (0·19 to 0·34)
	2016–30	76·14% (73·18 to 77·69)	92·26% (91·32 to 92·98)	2030	0·53 (0·37 to 0·73)	0·10 (0·06 to 0·16)
	2016–50	80·86% (78·35 to 82·60)	96·39% (96·01 to 96·81)	2050	0·32 (0·21 to 0·42)	0·12 (0·04 to 0·18)

Data are median (95% CrI). Cumulative incidence and mortality reductions were calculated as the percent by which incidence or mortality were reduced in each scenario relative to a counterfactual scenario (either the scenario without ART or the scenario without ART or VMMC). Negative values indicate increases in cumulative incidence or mortality. The incidence and proportion of new infections in people aged 15–24 years are shown for the end of each time horizon (ie, the year column). Median and 95% CrIs are reported for 250 of the best fitting parameter sets. ART=antiretroviral therapy. VMMC=voluntary medical male circumcision. CrI=credible interval.

**Figure 2:**
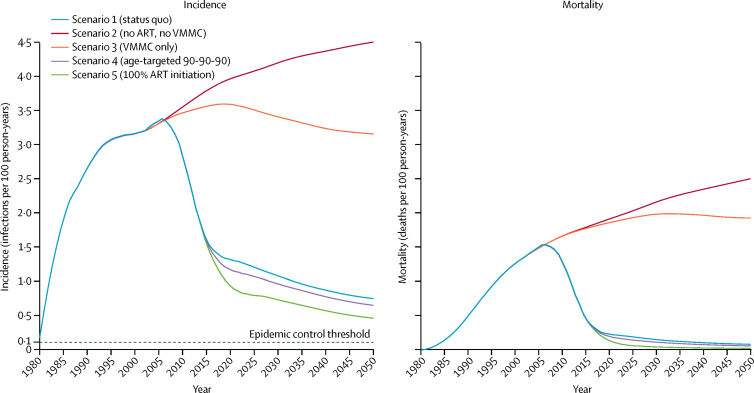
Incidence and mortality trajectories by scenarios 1–5 The curves are LOESS curves of 250 modelled simulations. The epidemic control threshold is an incidence of 0·1 infections per 100 person-years. ART=antiretroviral therapy. LOESS=locally weighted smoothing.

**Figure 3:**
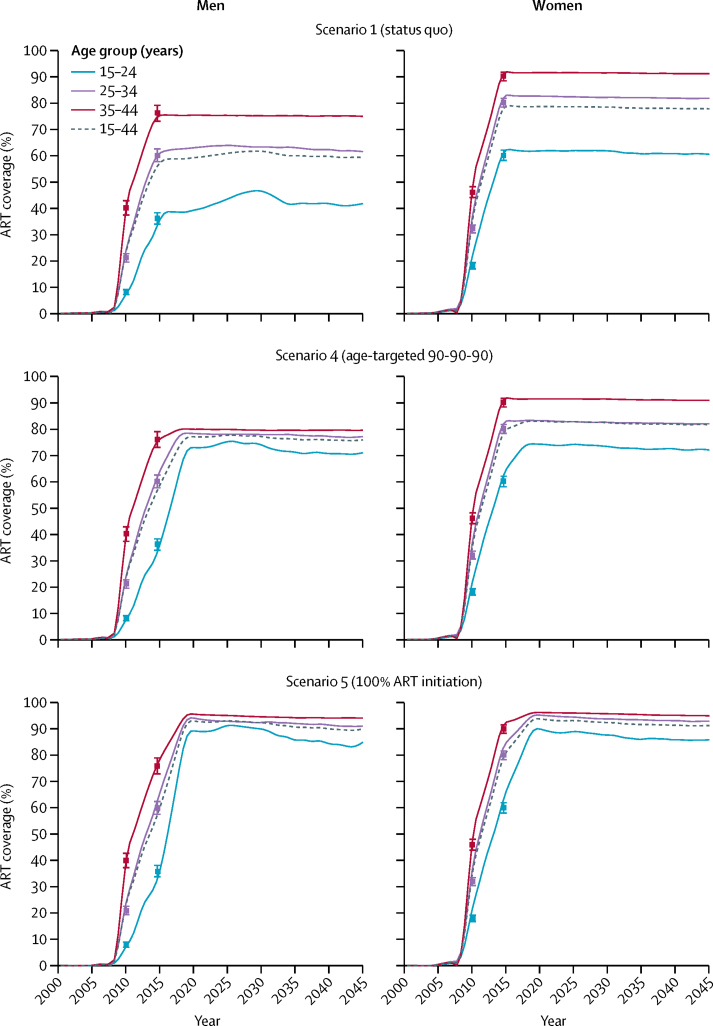
Modelled scale-up of ART by age group, sex, and scenario over time Data are point estimates (95% CI) from 2 survey years (2011 and 2016) overlaid.^14^, ^23^ ART=antiretroviral therapy.

Modelled incidence trajectories from 1980 to 2050 for each of the five scenarios are shown in figure 2, with numerical effect estimates shown in table 2. ART scale-up from the model output with point estimates of ART coverage from survey data is shown for scenario 1 (historical scale-up of ART and VMMC [status quo]), scenario 4 (age-targeted 90-90-90), and scenario 5 (100% ART initiation; figure 3). The historical scale-up of ART (scenario 1) reduced cumulative incidence by 43·57% (95% CrI 39·71–46·36) and cumulative mortality by 56·17% (54·06–58·92) among both men and women aged 15–49 years, between 2010 and 2016, compared with a counterfactual without ART (ie, scenario 3 for 2010–16). The historical scale-up of ART reduced HIV incidence to a larger extent among men than women in 2010–16, and reduced mortality more among women than men over the same time horizon (table 2). Under status quo (scenario 1), adult incidence declined to 1·09 infections (0·87–1·29) per 100 person-years by 2030, and to 0·74 infections (0·60–0·91) per 100 person-years by 2050. Under the age-specific and sex-specific 90-90-90 targeting of ART (scenario 4), adult HIV incidence and mortality improved only marginally beyond the effects of status quo ART (relative to scenario 3 for 2016–30; table 2).

The relative effect of VMMC on incidence was evaluated by comparing models with the scale-up of VMMC to 2016 levels with models without any VMMC scale-up, both in the absence of ART (scenario 3; table 2). VMMC contributed only modestly to short-term (2010–16) reductions in cumulative HIV incidence in individuals aged 15–49 years (relative to scenario 2 for 2010–16), although the effect of VMMC on incidence increased over longer time horizons and had a larger overall effect on HIV incidence reductions in men than women because of the direct benefit to circumcised men (scenario 3 relative to scenario 2 for 2016–50). In the absence of both ART and VMMC (scenario 2), HIV incidence in eSwatini continued to increase after its 2006 peak (figure 2), reaching 4·46 infections (95% CrI 3·64–5·23) per 100 person-years by 2050 (table 2). Under a scenario in which 100% ART initiation is assumed within an average of 6 months after infection (scenario 5), cumulative HIV mortality was reduced by 94·52% (94·05–94·93) and cumulative HIV incidence was reduced by 74·95% (72·23–76·84) from 2016 to 2030 (relative to scenario 3), with overall adult HIV incidence falling to 0·73 infections (0·55–0·92) per 100 person-years by 2030, and 0·46 infections (0·33–0·59) per 100 person-years by 2050. In no scenario did incidence reach the epidemic control target of 1 infection per 1000 person-years by 2030.

**Figure 4:**
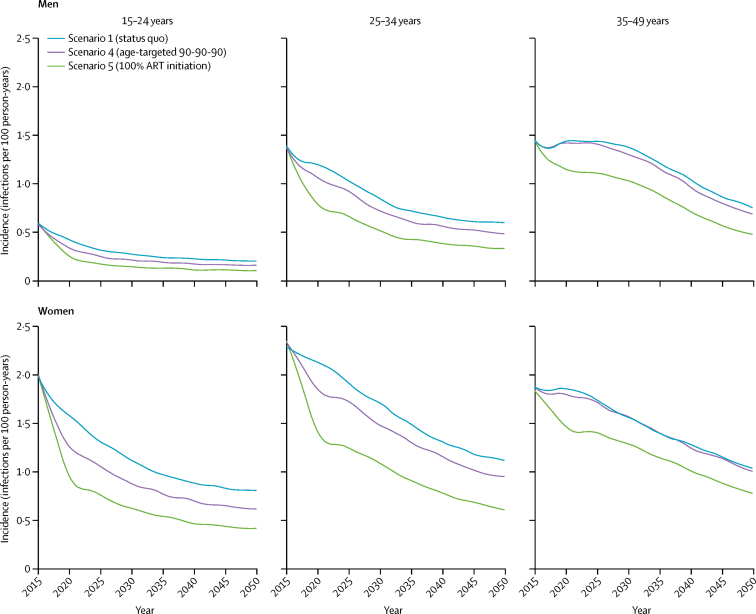
Incidence trajectories by ART scale-up scenario, age group, and sex from 2015 to 2050 The curves are LOESS curves of 250 modelled simulations. ART=antiretroviral therapy. LOESS=locally weighted smoothing.

**Figure 5:**
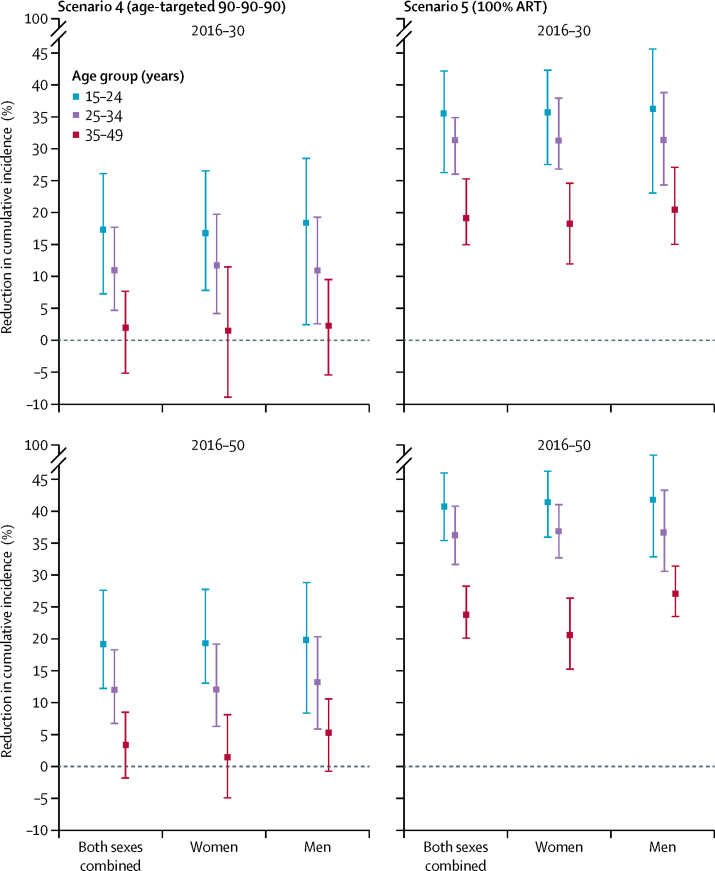
Reduction in cumulative HIV incidence for scenarios 4 and 5 relative to scenario 1 (status quo), by age and sex, during 2016–30 and 2016–50 Data are reduction (95% credible interval). Negative values indicate an increase in cumulative incidence. ART=antiretroviral therapy.

Incidence continued to decline beyond 2016 across all ART scale-up scenarios (scenarios 1, 4, and 5), with earlier and more dramatic declines in incidence among the youngest age groups (15–24 years and 25–34 years) because of the larger reduction in treatment gaps in those age groups than in older individuals (35–49 years; figure 4). Closing age and sex gaps in ART coverage beyond 2016 (scenario 4) had the greatest effect on reducing cumulative HIV incidence in young men and women aged 15–24 years (17·37% reduction, 95% CrI 7·33 to 26·12, for both sexes combined; 18·43%, 2·51 to 28·50, for men and 16·89%, 7·86 to 26·56, for women), relative to status-quo ART between 2016 and 2030, with minimal additional incidence reductions in adults aged 35–49 years (1·99%, −5·09 to 7·74; figure 5). Achieving 100% ART initiation (scenario 5) further closed treatment gaps in younger people living with HIV, resulting in even larger reductions in incidence than in the status-quo scenario (figure 5). Although scenarios under expanded ART coverage would initially increase the number of individuals on ART, these scenarios would further drive down incidence, resulting in fewer individuals requiring ART later on (appendix p 12).

The proportion of transmissions originating from individuals in the acute (within 3 months) and early (within 1 year) phases of HIV infection, modelled using scenario 1, varied over the course of the epidemic and increased following ART scale-up after 2005 (appendix p 13). By 2030, acute HIV infections accounted for 18·8% (95% CrI 13·7–22·4) of transmissions from men and 16·4% (12·8–21·8) of transmissions from women, and early infections made up 23·8% (18·8–28·0) of transmissions from men and 20·8% (16·0–26·0) from women. By 2030, HIV transmissions under the status-quo ART scale-up (scenario 1, with >80% ART coverage by 2030) originated from individuals who had either never been on ART (38·6%, 33·1–47·7), had been on ART but dropped out (24·7%, 20·2–29·6), or who were on ART but not virally suppressed (35·9%, 30·1–41·4); appendix p 14). Under 100% ART initiation (scenario 5, with 90% ART coverage) most transmissions originated from HIV-infected individuals who were on ART but not virally suppressed (57·0%, 46·1–67·1), followed by those who had never been on ART (29·4%, 18·2–44·3) and those who had dropped out from ART (13·7%, 8·7–17·7) (appendix p 14).

Reductions in HIV incidence had an additional effect of shifting both the age of acquisition and the age of transmission towards older ages over time in the ART era (appendix p 15). Under the status-quo scenario (scenario 1), the proportion of all new HIV acquisitions among individuals aged 15–49 years that occurred in those aged 15–24 years dropped from 0·39 (95% CrI 0·34–0·44) in 2016, to 0·26 (0·22–0·30) by 2030, and 0·24 (0·19–0·29) by 2050 (table 2). The 100% ART coverage scenario (scenario 5) produced the most dramatic ageing of the epidemic, with the proportion of new infections in the 15–24-years age group declining by 50%, from 0·40 (0·34–0·46) in 2016, to 0·21 (0·16–0·28) in 2030, after which a new equilibrium was established, and no further shift was observed (table 2). The ageing of the epidemic was fully attributable to interventions that reduced the force of infection (ie, ART and VMMC): no significant shifts in the age distribution of new infections occurred under the counterfactual scenario (scenario 2) without these interventions (table 2).

Modelled incidence was sensitive to uncertainty of four key parameters (acute duration in months, acute stage infectivity multiplier, time from infection to ART initiation, and ART efficacy), although varying these four parameters did not change the primary results of the analysis (appendix pp 16–22). Improving ART efficacy (≥98%) and reducing the time from infection until ART initiation (<2 months) resulted in HIV epidemic control by 2050 (appendix p 22).

## Discussion

To date, ART scale-up in eSwatini has dramatically reduced HIV-1 incidence and mortality in individuals aged 15–49 years; yet at current ART coverage levels incidence will remain above 1 infection per 100 person-years for decades to come unless further progress is made. Results from our model, EMOD, are consistent with data from eSwatini suggesting a substantial decline in directly estimated HIV incidence, from 2·7 infections per 100 person-years in 2011, to 1·4 infections per 100 person-years in 2016.^15^, ^25^ ART scale-up has, furthermore, contributed to large declines in HIV mortality in eSwatini, a more than 50% decline from 2010 to 2016. Closing current treatment coverage gaps to reach 90-90-90 evenly across age and sex groups by 2020 will add marginally to future declines in adult HIV incidence but will have a large effect in reducing incidence among younger age groups (15–24 years), a result of filling the gap in ART coverage among younger people living with HIV.

Despite dramatic reductions in incidence and mortality, our results suggest that ART scale-up alone will not be enough to achieve epidemic control as previous models have suggested,^7^, ^28^ both due to operational constraints of achieving high coverage and the epidemiological challenges of continuing to bring down transmission with ART as the primary intervention. In the HPTN 071 PopART community-randomised trial of universal test and treat, for example, less than half of HIV-infected, ART-naive men and women referred to HIV care initiated ART within 6 months, with just over half initiating by 12 months.^29^ In a randomised controlled trial testing various linkage-to-care modalities in South Africa and Uganda, although most HIV-positive individuals were linked to care, only half achieved viral load suppression 9 months after the intervention.^30^ The time interval from seroconversion until diagnosis, treatment, and viral suppression remains an uncertain but important interval over which persistent transmission occurs in the era of high overall ART coverage. The variability in this delay is likely to depend on several population-specific risk factors, with the potential that individuals most likely to transmit HIV soon after infection might be least likely to test, link to care, and initiate ART.^13^

Mathematical models of universal test and treat have historically overestimated the population-level effect of treatment scale-up on incidence reductions.^7^, ^28^ The reasons for this include a lack of age and sex structuring in modelled treatment scale-up, overly simplistic assumptions around sexual mixing patterns, and unrealistic assumptions on ART scale-up. Our model overcomes these limitations by explicitly calibrating a cascade of care to reflect gaps in the historical scale-up of ART by age and sex and by including empirical age-specific sexual mixing patterns. As a result, the attenuating effects of age-specific and sex-specific disparities in the coverage of treatment and prevention are incorporated into our projections of treatment effect. Such disparities in coverage are common across sub-Saharan African settings^10^, ^12^ and result from age-cohort effects, survival effects, and age-specific and sex-specific differences in access to, and uptake of, treatment and care.

Although universal test and treat remains an important component of combination HIV prevention, substantial uncertainty has surrounded the extent to which it can reduce incidence.^31^ The population-level effectiveness of universal test and treat depends on a number of contextual epidemiological factors, including whether treatment reaches those with the greatest potential for onward transmission and whether ART is started early enough to subvert onward transmission. Individuals with the greatest potential for transmission (younger men, mobile populations, and higher-risk individuals) tend to have lower prevalence of viral suppression.^11^, ^12^ Countries that have achieved the 90-90-90 goals continue to face the challenge of engaging HIV-infected individuals at high risk of transmission.

Our results suggest that early and acute HIV infections play a large role in the transmission of HIV as overall treatment coverage expands, consistent with previous models.^32^, ^33^ Transmissions that occur soon after infection, before an individual gains awareness of their status and initiates treatment, have the potential to attenuate the effect of test and treat given the increased risk of transmission during that time. There is considerable debate, however, as to the extent to which transmission during early and acute HIV infections attenuates incidence reduction from universal test and treat.^31^, ^34^ On the basis of our sensitivity analysis of ART efficacy and time to HIV diagnosis, epidemic control (incidence <1 infection per 1000 person-years) was only achieved by 2050 with cross-sectional ART coverage greater than 90%, ART efficacy of 98% or more, and less than 2 months between HIV acquisition and ART initiation among those who take up treatment, an overly optimistic and highly unrealistic treatment goal. Our results confirm that even under high rates of testing and linkage to care, HIV transmission can persist at endemic levels and above control thresholds because of demographic gaps in treatment coverage, the contribution of early and acute HIV infections, the fraction of individuals on ART who are unsuppressed, and dropout from ART.

Our model results highlight sex asymmetries in the short-term effects of ART on incidence and mortality. Higher treatment coverage in women than in men, common across epidemic settings, reduces mortality more among women and incidence more among men. Gaps in treatment coverage alone account for sex-specific disparities in incidence reductions observed in our study and are likely to play out across sub-Saharan African settings. Sex asymmetries in incidence declines have been observed in population-based cohort studies after ART scale-up, including in the Rakai community cohort study, in which incidence declines among men (adjusted incidence rate ratio 0·46, 95% CrI 0·29–0·73) were larger than those among women (adjusted incidence rate ratio 0·68, 0·50–0·94),^5^ and the Africa Health Research Institute cohort, in which women's incidence has not declined to the same extent as men's.^8^

Our analysis has some important limitations. We did not simulate additional VMMC scale-up beyond 2016 levels. Circumcision coverage in eSwatini is among the lowest of high-priority countries in sub-Saharan Africa and is not likely to reach current targets,^35^ making it difficult to forecast what further gains will be achieved. The effect of VMMC on HIV incidence in our model should thus be interpreted under the assumption that male circumcision coverage stays at 2016 levels, which is likely to underestimate future coverage. Similarly, we did not model the layered effects of other HIV prevention modalities, including the roll-out of pre-exposure prophylaxis. Finally, our results might only be generalisable to settings with similar epidemic patterns and treatment scale-up dynamics.

Continuing to close gaps in ART coverage by age, sex, and risk profile has the potential to further drive down incidence in generalised epidemics, especially among younger cohorts. Continuing with expanded treatment policies—such as those undertaken by eSwatini and other countries that have scaled up ART in historically low-coverage demographic groups—will not only continue to save lives and prevent infections but will also avert future treatment costs. However, even under the most ideal of scenarios, ART coverage will be insufficient to bring infections below epidemic control levels in the world's most intense HIV epidemic settings. Ending HIV will thus require the expansion of additional HIV prevention modalities in addition to aggressive treatment coverage.

## Data sharing

All data used for model calibration are publicly available and can be found in a summary table in the appendix (pp 1, 2). For further exploration of model output from this study please visit the eSwatini HIV model dashboard.
