# Supplementary material for: The effect of 90-90-90 on HIV-1 incidence and mortality in eSwatini: a mathematical modelling study
Source: Lancet HIV. 2020 Feb 13;7(5):e348–58. doi: 10.1016/S2352-3018(19)30436-9 (PMC7221345; doi:10.1016/S2352-3018(19)30436-9)
Supplement: Supplementary appendix [file mmc1.pdf]

THE LANCET
HIV

Supplementary appendix

This appendix formed part of the original submission and has been peer reviewed. We post it as supplied by the authors.

Supplement to: Akullian A, Morrison M, Garnett GP, et al. The effect of 90-90-90 on HIV-1 incidence and mortality in eSwatini: a mathematical modelling study. *Lancet HIV* 2020; published online Feb 13. https://doi.org/10.1016/S2352-3018(19)30436-9.

Appendix

Table A1. HIV prevalence data by age and sex from three population-based surveys (SDHS 2007, SHIMS 2011, and PHIA 2016). Note that the youngest age-group in 2011 was 18-19 year-olds.

Year	Sex	Age	Count	Prevalence (%)	95% lb	95% ub	Source
2007	Male	15 - 20	1272	1.9	1.2	2.7	SDHS 2007
2007	Male	20 - 25	779	12.4	10.2	14.8	SDHS 2007
2007	Male	25 - 30	553	27.8	24.1	31.6	SDHS 2007
2007	Male	30 - 35	380	43.7	38.8	48.7	SDHS 2007
2007	Male	35 - 40	321	44.9	39.5	50.4	SDHS 2007
2007	Male	40 - 45	230	40.7	34.4	47.1	SDHS 2007
2007	Male	45 - 50	229	27.9	22.3	33.9	SDHS 2007
2007	Male	50 - 55	106	28.3	20.2	37.2	SDHS 2007
2007	Male	55 - 60	70	17.4	9.5	27.0	SDHS 2007
2007	Male	60 - 65	227	13.3	9.2	18.0	SDHS 2007
2007	Female	15 - 20	1151	10.1	8.4	11.9	SDHS 2007
2007	Female	20 - 25	922	38.4	35.3	41.6	SDHS 2007
2007	Female	25 - 30	648	49.2	45.4	53.0	SDHS 2007
2007	Female	30 - 35	536	45.2	41.0	49.4	SDHS 2007
2007	Female	35 - 40	441	37.7	33.2	42.3	SDHS 2007
2007	Female	40 - 45	382	27.9	23.5	32.5	SDHS 2007
2007	Female	45 - 50	342	21.4	17.2	25.9	SDHS 2007
2007	Female	50 - 55	144	24.3	17.7	31.6	SDHS 2007
2007	Female	55 - 60	102	9.6	4.7	16.0	SDHS 2007
2007	Female	60 - 65	342	7	4.5	9.9	SDHS 2007
2011	Male	18 - 20	997	1	0.5	1.7	SHIMS 2011
2011	Male	20 - 25	2093	7	5.9	8.1	SHIMS 2011
2011	Male	25 - 30	1679	21	19.1	23.0	SHIMS 2011
2011	Male	30 - 35	1267	37	34.4	39.7	SHIMS 2011
2011	Male	35 - 40	993	47	43.9	50.1	SHIMS 2011
2011	Male	40 - 45	726	46	42.4	49.6	SHIMS 2011
2011	Male	45 - 50	571	43	39.0	47.1	SHIMS 2011
2011	Female	18 - 20	989	14	11.9	16.2	SHIMS 2011
2011	Female	20 - 25	2488	31	29.2	32.8	SHIMS 2011
2011	Female	25 - 30	1925	47	44.8	49.2	SHIMS 2011
2011	Female	30 - 35	1361	54	51.3	56.6	SHIMS 2011
2011	Female	35 - 40	1208	49	46.2	51.8	SHIMS 2011
2011	Female	40 - 45	976	40	36.9	43.1	SHIMS 2011
2011	Female	45 - 50	893	32	29.0	35.1	SHIMS 2011
2011	Male	15 - 20	540.72	3.9	2.4	5.7	SHIMS 2011
2016	Male	20 - 25	901.2	4.2	3.0	5.6	SHIMS2 2016
2016	Male	25 - 30	901.2	13.3	11.2	15.6	SHIMS2 2016

2016	Male	30 - 35	675.9	28.1	24.8	31.5	SHIMS2 2016
2016	Male	35 - 40	540.72	41.9	37.8	46.1	SHIMS2 2016
2016	Male	40 - 45	405.54	43.3	38.5	48.1	SHIMS2 2016
2016	Male	45 - 50	180.24	48.8	41.5	56.1	SHIMS2 2016
2016	Male	50 - 55	135.18	41.9	33.7	50.3	SHIMS2 2016
2016	Male	55 - 60	135.18	31.8	24.2	39.9	SHIMS2 2016
2016	Male	60 - 65	90.12	31.9	22.7	41.8	SHIMS2 2016
2016	Female	15 - 20	642.8	7.2	5.3	9.3	SHIMS2 2016
2016	Female	20 - 25	1414.2	20.9	18.8	23.1	SHIMS2 2016
2016	Female	25 - 30	964.2	37.5	34.5	40.6	SHIMS2 2016
2016	Female	30 - 35	771.36	50.7	47.2	54.2	SHIMS2 2016
2016	Female	35 - 40	771.36	54.2	50.7	57.7	SHIMS2 2016
2016	Female	40 - 45	578.52	51.9	47.8	56.0	SHIMS2 2016
2016	Female	45 - 50	578.52	42.3	38.3	46.3	SHIMS2 2016
2016	Female	50 - 55	257.12	36.1	30.3	42.1	SHIMS2 2016
2016	Female	55 - 60	257.12	29.5	24.1	35.2	SHIMS2 2016
2016	Female	60 - 65	192.84	22.3	16.7	28.4	SHIMS2 2016

Table A2. Select model parameters used to fit the EMOD transmission model to survey data on HIV prevalence, ART coverage, and population demgraphics from the Kingdom of eSwatini. Median and interquartile ranges (IQRs) reported for all dynamic parameters used in the calibration process (N=24) from 250 best-fitting parameter sets. A full description of all parameters and references available is at: http://idmod.org/docs/hiv/parameter-configuration.html

Parameter	Description	Dynamic	Static value / fitted median (IQR)	Source
Acute_Duration_In_Months	The time since infection, in months, over which the Acute_Stage_Infectivity_Multiplier is applied to coital acts occurring in that time-period.	no	3	[1]
Acute_Stage_Infectivity_Multiplier	Multiplier acting on Base_Infectivity to determine the per-act transmission probability of an individual during acute stage	no	26	[1]
AIDS_Duration_In_Months	The length of time, in months, prior to an AIDS-related death over which the AIDS_Stage_Infectivity_Multiplier is applied	no	9	[1]
AIDS_Stage_Infectivity_Multiplier	Multiplier acting on Base_Infectivity to determine the per-act transmission probability of an individual during AIDS stage	no	4.5	[1]
ART_CD4_at_Initiation_Saturating_Reduction_in_Mortality	The duration from ART enrollment to on-ART HIV-caused death increases with CD4 at ART initiation up to a threshold determined by this parameter value.	no	350	
ART_dropout	Exponentially distributed mean number of days from ART initiation until ART dropout	no	7300	
ART_Link_Max	The right asymptote for the sigmoid trend of probability of ART linkage (given eligibility) over time.	yes	0.952 (0.948 - 0.955)	
ART_Link_Mid	The time of the inflection point in the sigmoid trend of probability of ART linkage (given eligibility) over time.	yes	2010.7 (2010.4 - 2010.9)	
ART_link_Min	The left asymptote for the sigmoid trend of probability of ART linkage (given eligibility) over time.	no	0	
ART_link_Rate	The slope of the inflection point in the sigmoid trend of probability of ART linkage over time. A Rate of 1 sets the slope to a 25% change in probability per year.	no	1	

ART_Viral_Suppression_Multiplier	Multiplier acting on Base_Infectivity to determine the per-act transmission probability of an individual on ART. Less-than-perfect (<100%) reduction in risk is attributed to sub-optimal adherence, drug resistance, and delay in viral load suppression from ART initiation.	no	0.08	[2]
Base_Infectivity	The probability of transmission when none of the transmission multipliers apply to a coital act (or when all multipliers are set to 1).	yes	0.00233 (0.00231 - 0.00234)	[3]
CD4_At_Death_LogLogistic_Heterogeneity	The inverse shape parameter of a Weibull distribution that represents the at-death CD4 cell count.	no	0.7	
CD4_At_Death_LogLogistic_Scale	The scale parameter of a Weibull distribution hat represents the at-death CD4 cell count.	no	2.96	
CD4_Post_Infection_Weibull_Heterogeneity	The inverse shape parameter of a Weibull distribution that represents the post-acute-infection CD4 cell count.	no	0.2756	
CD4_Post_Infection_Weibull_Scale	The scale parameter of a Weibull distribution that represents the post-acute-infection CD4 cell count.	no	560.43	
Circumcision_Reduced_Acquire	The reduction of susceptibility to HIV by voluntary male medical circumcision (VMMC)	no	0.6	[4-6]
Coital_Act_Rate	Number of coital acts per day for all relationships except commercial ones	no	0.33	
Coital_Act_Rate_Commercial	Number of coital acts per day for commercial relationships	no	0.002739726	
Coital_Dilution_Factor_2_Partners	The multiplicative reduction in the coital act rate for all relationship types when an individual has exactly two current partners. Represents coital dilution.	no	0.75	
Coital_Dilution_Factor_3_Partners	The multiplicative reduction in the coital act rate for all relationship types when an individual has exactly three current partners. Represents coital dilution.	no	0.6	
Coital_Dilution_Factor_4_Plus_Partners	The multiplicative reduction in the coital act rate for all relationship types when an individual has exactly three current partners. Represents coital dilution.	no	0.45	

Commercial_Condom_Max	The maximum asymptote for commercial relationships	no	0.85
Commercial_Condom_Mid	The year of the inflection point for commercial relationships	no	1999.5
Commercial_Condom_Min	The minimum asymptote of the probability of condom use per coital act for informal relationships for commercial relationships	no	0.5
Commercial_Condom_Rate	The rate proportional to the slope at the inflection point for commercial relationships	no	1
Commercial_Form_Rate	Exponentially distributed mean number new relationships formed per day for commercial relationships	no	0.15
Condom_Transmission_Blocking_Probability	The per-act multiplier of the transmission probability when a condom is used	no	0.8
Days_Between_Symptomatic_And_Death_Weibull_Heterogeneity	The time between the onset of AIDS symptoms and death is sampled from a Weibull distribution; this parameter governs the heterogeneity (inverse shape) of the Weibull.	no	0.5
Days_Between_Symptomatic_And_Death_Weibull_Scale	The time between the onset of AIDS symptoms and death is sampled from a Weibull distribution; this parameter governs the scale of the Weibull.	no	618.34
Delay_Period_Mean	Delay from HIV infection until ART initiation for future ART scale-up scenarios, post 2016 (in days).	no	180
HIV_Adult_Survival_Scale_Parameter_Intercept	Determines the intercept of the scale parameter for the Weibull distribution used to determine HIV survival time. Survival time with untreated HIV infection depends on the age of the individual at the time of infection, and is drawn from a Weibull distribution with shape parameter (see HIV_Adult_Survival_Shape_Parameter) and scale parameter. The scale parameter is allowed to vary linearly with age as follows λ = HIV_Adult_Survival_Scale_Parameter_Intercept + HIV_Adult_Survival_Scale_ Parameter_Slope * Age (in years).	no	21.182

HIV_Adult_Survival_Scale_Parameter_Slope	This parameter determines the slope of the scale parameter for the Weibull distribution used to determine HIV survival time.	no	-0.2717
HIV_Adult_Survival_Shape_Parameter	This parameter determines the shape of the Weibull distribution used to determine age-dependent survival time for individuals infected with HIV.	no	2
HIV_Age_Max_for_Adult_Age_Dependent_Survival	Survival time with untreated HIV infection depends on the age of the individual at the time of infection, and is drawn from a Weibull distribution with shape parameter and scale parameters (See HIV_Adult_Survival_Scale_Parameter_Intercept, HIV_Adult_Survival_Scale_ Parameter_Slope, and HIV_Adult_Survival_Shape_Parameter). Although the scale parameter for survival time declines with age, it cannot become negative. To avoid negative survival times at older ages, this parameter, HIV_Age_Max_for_Adult_Age_Dependent _Survival, determines the age beyond which HIV survival is no longer affected by further aging.	no	50
HIV_Age_Max_for_Child_Survival_Function	The maximum age at which an individual's survival will be fit to the child survival function. If the value of this parameter falls between zero and the age of sexual debut, model results are not sensitive to this parameter as there is no mechanism for children to become infected between infancy and sexual debut.	no	15
HIV_Child_Survival_Rapid_Progressor_Fraction	The proportion of HIV-infected children who are rapid HIV progressors.	no	0.57
HIV_Child_Survival_Rapid_Progressor_Rate	The exponential decay rate, in years, describing the distribution of HIV survival for children who are rapid progressors.	no	1.52
HIV_Child_Survival_Slow_Progressor_Scale	The Weibull scale parameter describing the distribution of HIV survival for children who are slower progressors.	no	16

HIV_Child_Survival_Slow_Progressor_Shape	The Weibull shape parameter describing the distribution of HIV survival for children who are slower progressors.	no	2.7
Informal_Condom_Max	The maximum asymptote for informal relationships	yes	0.337 (0.321 - 0.355)
Informal_Condom_Mid	The year of the inflection point for informal relationships	yes	1992.6 (1992.2 - 1992.9)
Informal_Condom_Min	The minimum asymptote of the probability of condom use per coital act for informal relationships	no	0
Informal_Condom_Rate	The rate proportional to the slope at the inflection point for informal relationships		3.003 (2.941 - 3.076)
Informal_Form_Rate	Exponentially distributed mean number new relationships formed per day for informal relationships	yes	0.00146 (0.00134 - 0.00155)
Male_To_Female_Relative_Infectivity_Multiplier_Old	An array of scale factors governing the susceptibility of females relative to males, by age ≥ 25	yes	2.844 (2.727 - 2.958)
Male_To_Female_Relative_Infectivity_Multiplier_Young	An array of scale factors governing the susceptibility of females relative to males, by age < 25	yes	4.894 (4.747 - 5.041)
Marital_Condom_Max	The maximum asymptote for marital relationships	yes	0.218 (0.207 - 0.231)
Marital_Condom_Mid	The year of the inflection point for marital relationships	yes	2001.8 (2001.5 - 2002.1)
Marital_Condom_Min	The minimum asymptote of the probability of condom use per coital act for informal relationships for marital relationships	no	0
Marital_Condom_Rate	The rate proportional to the slope at the inflection point for marital relationships	yes	2.407 (2.252 - 2.524)
Marital_Form_Rate	Exponentially distributed mean number new relationships formed per day for marital relationships	yes	0.00046 (0.00044 - 0.0005)
Maternal_Infection_Transmission_Probability	The probability of transmission of infection from mother to infant at birth.		0.3
Maternal_Transmission_ART_Multiplier	The maternal transmission multiplier for on-ART mothers.	no	0.03334

preART_Link_Max	The right asymptote for the sigmoid trend of probability of preART linkage (given eligibility) over time.	yes	0.807 (0.783 - 0.829)
preART_Link_Mid	The time of the inflection point in the sigmoid trend of probability of preART linkage (given eligibility) over time.	yes	1995.7 (1995.1 - 1996.4)
preART_link_Min	The left asymptote for the sigmoid trend of probability of preART linkage (given eligibility) over time.	yes	0.00325 (0 - 0.03031)
preART_link_Rate	The slope of the inflection point in the sigmoid trend of probability of preART linkage over time. A Rate of 1 sets the slope to a 25% change in probability per year.	no	1
Proportion_Low_Risk	Proportion of the intial population that is low risk	yes	0.73 (0.721 - 0.742)
Seed_Year	Year in which the epidemic is seeded into high risk groups	yes	1982.7 (1982.4 - 1983.2)
Sexual_Debut_Age_Female_Weibull_Heterogeneity	The inverse shape of the Weibull distribution for female debut age.	yes	0.309 (0.293 - 0.322)
Sexual_Debut_Age_Female_Weibull_Scale	The scale term of the Weibull distribution for female debut age.	yes	16.302 (16.166 - 16.396)
Sexual_Debut_Age_Male_Weibull_Heterogeneity	The inverse shape of the Weibull distribution for male debut age.	yes	0.042 (0.04 - 0.05)
Sexual_Debut_Age_Male_Weibull_Scale	The scale term of the Weibull distribution for male debut age.	yes	17.499 (17.357 - 17.699)
Sexual_Debut_Age_Min	The minimum age at which individuals become eligible to form sexual relationships.	no	13
Transitory_Condom_Max	The maximum asymptote for transitory relationships	yes	0.103 (0.089 - 0.117)
Transitory_Condom_Mid	The year of the inflection point for transitory relationships	yes	1996.7 (1996.1 - 1997)
Transitory_Condom_Min	The minimum asymptote of the probability of condom use per coital act for informal relationships for transitory relationships	no	0
Transitory_Condom_Rate	The rate proportional to the slope at the inflection point for transitory relationships	yes	2.998 (2.878 - 3.106)

Transitory_Form_Rate	Exponentially distributed mean number new relationships formed per day for transitory relationships	no	0.001047839
Transitory_Weibull_Heterogeneity	Inverse of the Weibull shape (1/kappa) parameter of relationship duration in years for transitory relationships	no	0.833333333
Transitory_Weibull_Scale	Weibull scale parameter of relationship duration in years for transitory relationships.	no	0.956774771

Figure A1. Best-fitting HIV prevalence trajectories (N=250) by sex, five-year age group, and year, with survey prevalence point estimates and 95% confidence intervals from three surveys [SDHS (2007), SHIMS (2011), and PHIA (2016)] overlaid.

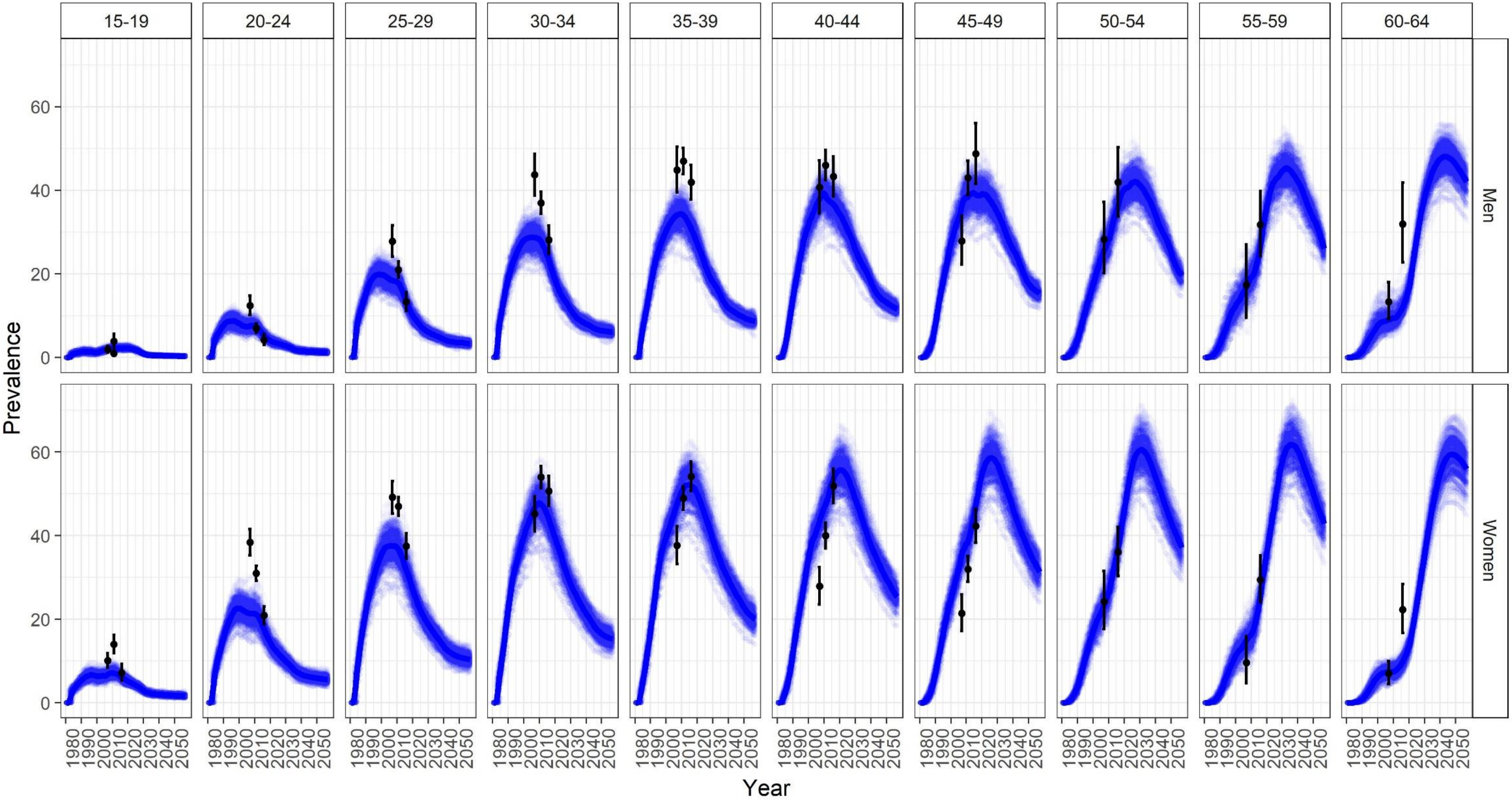

Modelled effect estimates

We used four metrics to quantify the population-level effectiveness of each scenario: 1) annual incidence, 2) annual mortality, 3) attributable reduction in cumulative incidence, and 4) percent reduction in cumulative mortality. Annual incidence was calculated as the number of new infections over a one-year period divided by the number of uninfected individuals at the mid-year, reported per 100 person-years (py). Annual mortality was calculated as the number of HIV/AIDS deaths in a year divided by the number of individuals alive at the mid-year, reported per 100 py. The attributable reduction in cumulative incidence (AR) between an intervention scenario *i* and a counterfactual scenario *j* was calculated as the difference in cumulative incidence between scenarios *j* and *i* divided by the cumulative incidence in scenario *j*,

$$AR_{ij} = 1 - \frac{\frac{\sum_{year=n}^{m} \mathrm{ninf}_{i,year}}{\sum_{year=n}^{m} \mathrm{py}_{i,year}}}{\frac{\sum_{year=n}^{m} \mathrm{ninf}_{j,year}}{\sum_{year=n}^{m} \mathrm{py}_{j,year}}}$$

where cumulative incidence is the sum over a time horizon (start year = n, end year = m) of the number of yearly new infections (ninf_{year}) occurring divided by the sum over the same time horizon (*n-m)* of the number of yearly person-years(py_{year}) among uninfected individuals. Percent reduction in mortality was estimated similarly, with the denominator being all person-years lived regardless of HIV status. LOESS (Locally weighted smoothing) was used to smooth and plot the time series of each metric from 250 model parametrizations. Credible intervals (CrI) for all effect estimates are calculated as 95% quantiles of the 250 best-fitting parameter sets.

Figure A2. Number of individuals on ART by age and sex under three ART scale-up scenarios. The intersection of curves indicates the year in which a more aggressive ART scale-up scenario results in fewer PLHIV on treatment.

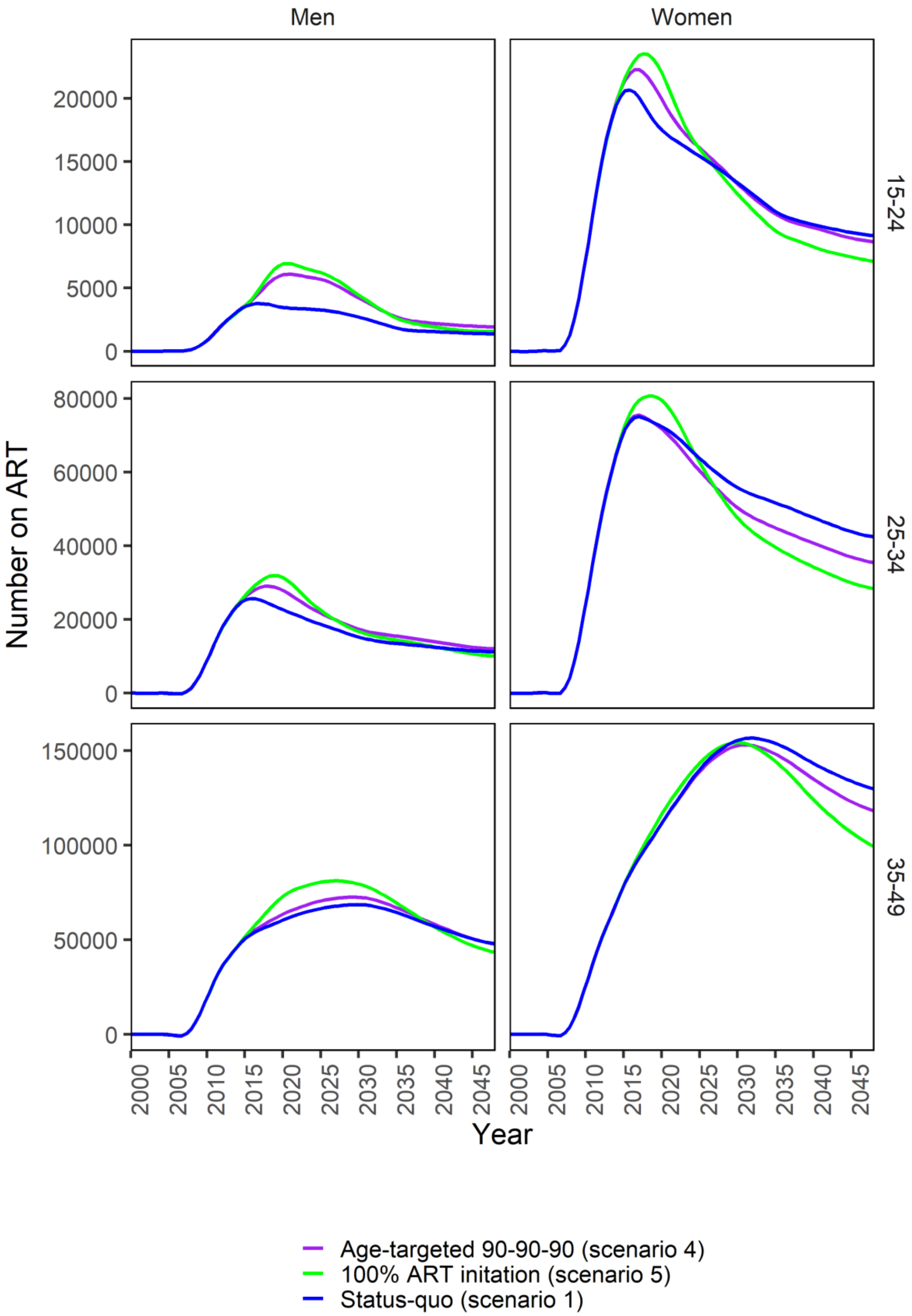

Figure A3. Proportion of transmissions from index cases in the acute (< 3 months) and early (< 1 year) stages of infection, by sex and year, under the status-quo scenario (scenario 1). Loess curves (dark lines) of 250 best-fitting simulations overlaid.

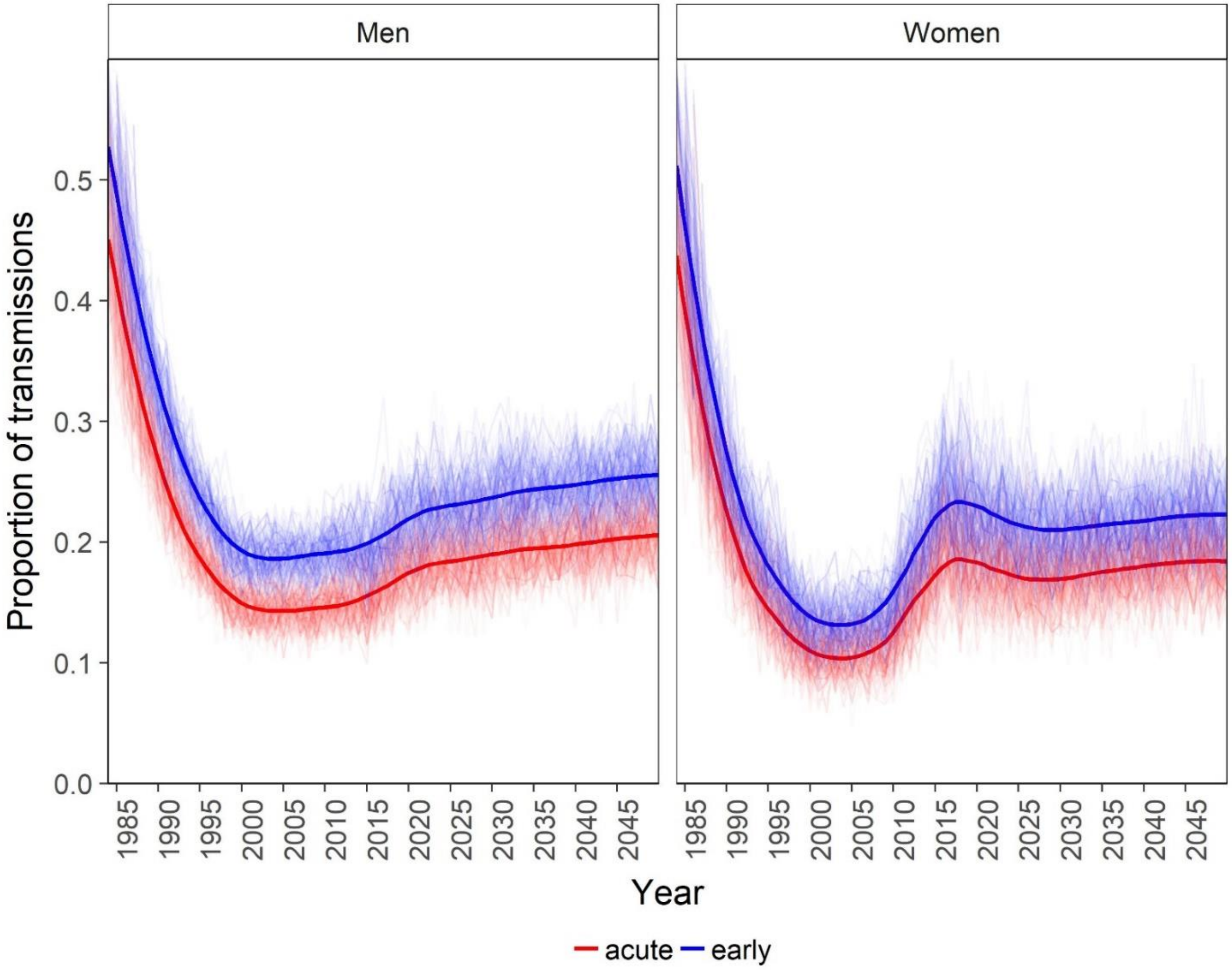

Figure A4. Proportion of transmissions over time by ART state of transmitter (never initiated ART, currently on ART, or dropped out from ART) for two modelling scenarios. ART coverage among adults 15-49 (% of HIV infected currently on ART) shown in dashed black line.

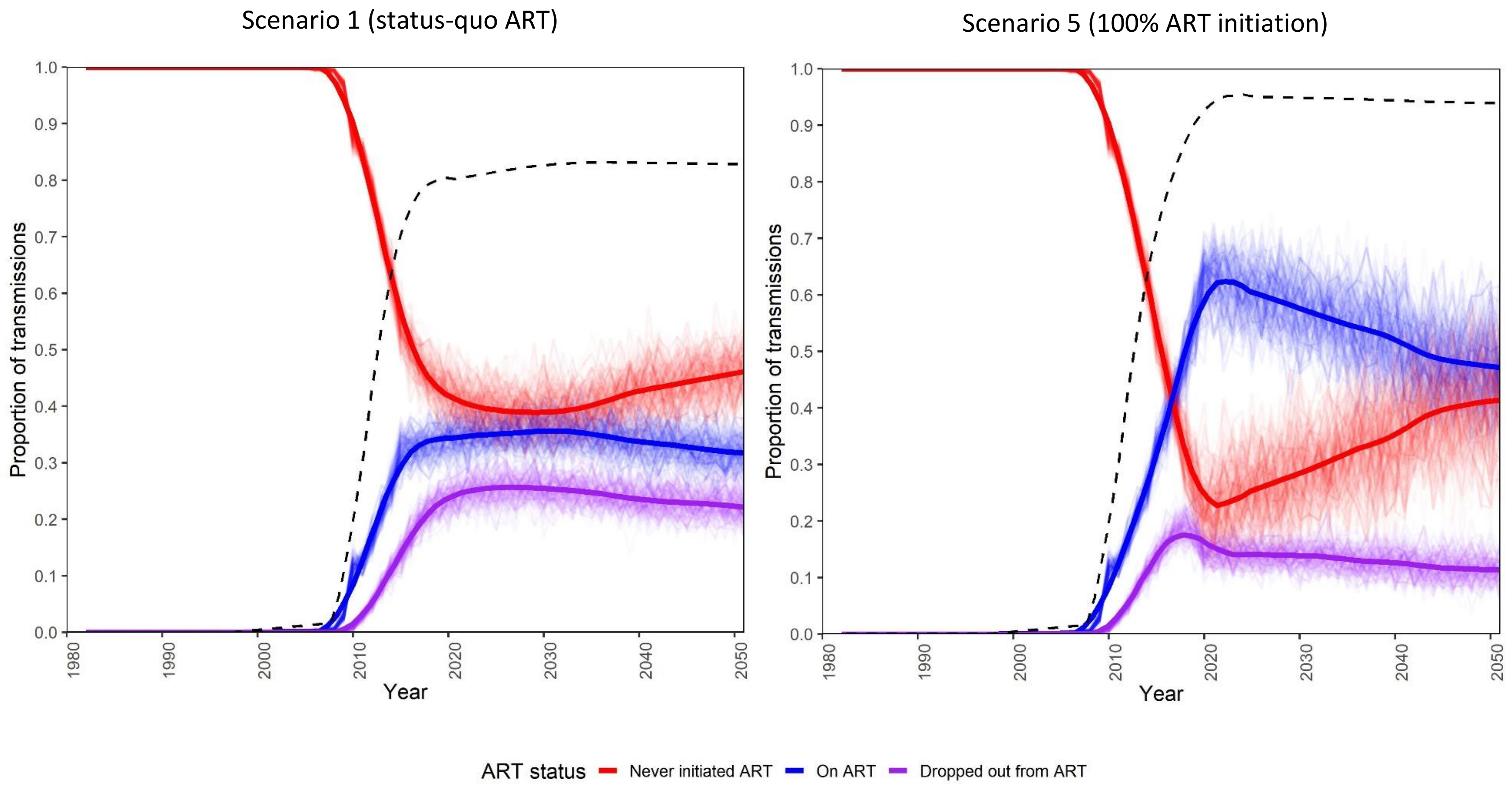

Figure A5. Age-distribution of HIV-1 acquisitions (a) and transmissions (b) by sex and year (2005 – 2050) under the status-quo scenario (scenario 1). The scale-up of ART over time shifted both the age of acquisition and age of transmission older. The phenomenon of the aging HIV-1 epidemic has been observed in age-shifts in HIV prevalence across sub-Saharan Africa (SSA) [49,50], (including in the Kingdom of Eswatini[51]) over the past two decades. Age-specific shifts in HIV incidence over time are the result of dynamic epidemic processes that lead to differential changes in the transmission rate by age and sex. These processes, which may be acting simultaneously, include aging HIV prevalence (the source of transmissions), age-specific targeting of HIV prevention to younger cohorts, and delayed age at infection with a declining force of infection. While most of the empirical data on epidemic aging is on shifting prevalence, our results confirm previous model-based evidence of shifting age-distribution of HIV incidence [52].

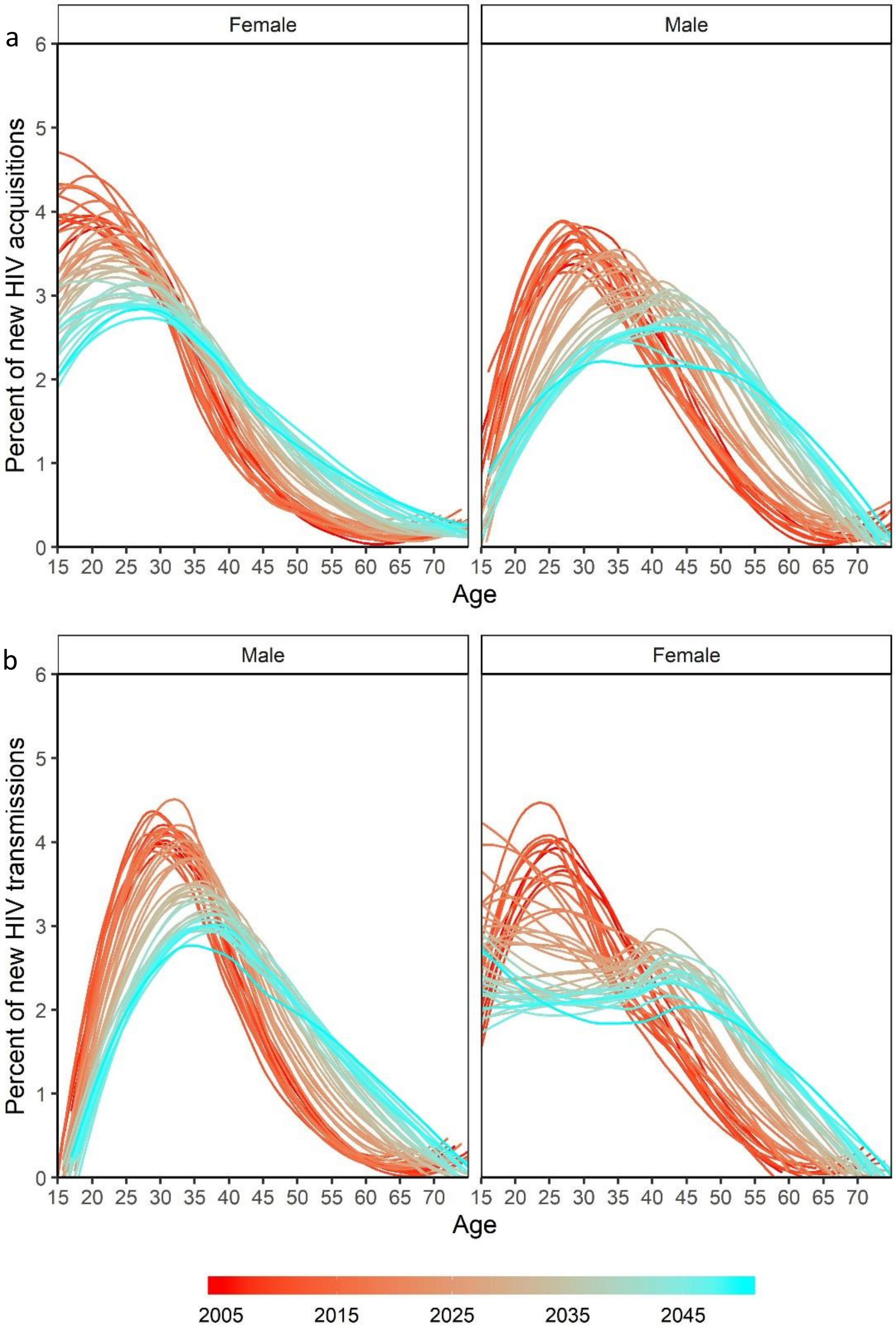

Sensitivity analyses:

We conducted sensitivity analyses around the contribution of four key parameters, both univariately and bivariately, to our incidence estimates for scenario 1:

a. Acute_Duration_In_Months
b. Acute_Stage_Infectivity_Multiplier
c. Delay_Period_Mean (time from infection to ART initiation in the UTT era)
d. ART_Viral_Suppression_Multiplier

1) Acute stage parameters

The primary sources of uncertainty in our model arise from the extent to which unsuppressed individuals contribute to onward transmission in the era of test and treat. Estimating this quantity has been challenging, as it is governed by parameters with high levels of uncertainty and disagreement in the literature, including the duration of acute and early HIV infection (literature modelled parameter ranges from 2 - 5 months[7,8]) and the elevated infectiousness during those periods (literature modelled parameter ranges from a multiplier of 10 - 30[7-9]). Sensitivity analyses around the contribution of four key parameters to our incidence estimates for scenario 1 in our model are shown below. Though the overall shape of the incidence curves over time remained relatively the same, results of this analysis highlight the sensitivity of our incidence (age 15-49) estimates to uncertainty of both acute stage infectivity multiplier and acute stage duration. We tested five values for acute stage infectivity multiplier (ranging from 10 - 30 by 5-unit increments), and five values of acute stage duration in months (ranging from 1 - 5 months by 1-month increments).

Figure A6. Sensitivity of HIV incidence over time (baseline scenario) varying two key parameters: a) acute stage duration in months, b) acute stage infectivity multiplier (relative to the chronic stage), and c) the bivariate sensitivity over combinations of both parameter estimates, evaluated at four time-points (2016, 2020, 2030, and 2050). The parameter values used in the final calibrated model noted with a (+). In each univariate sensitivity analysis, all other parameters are held according to the original calibration.

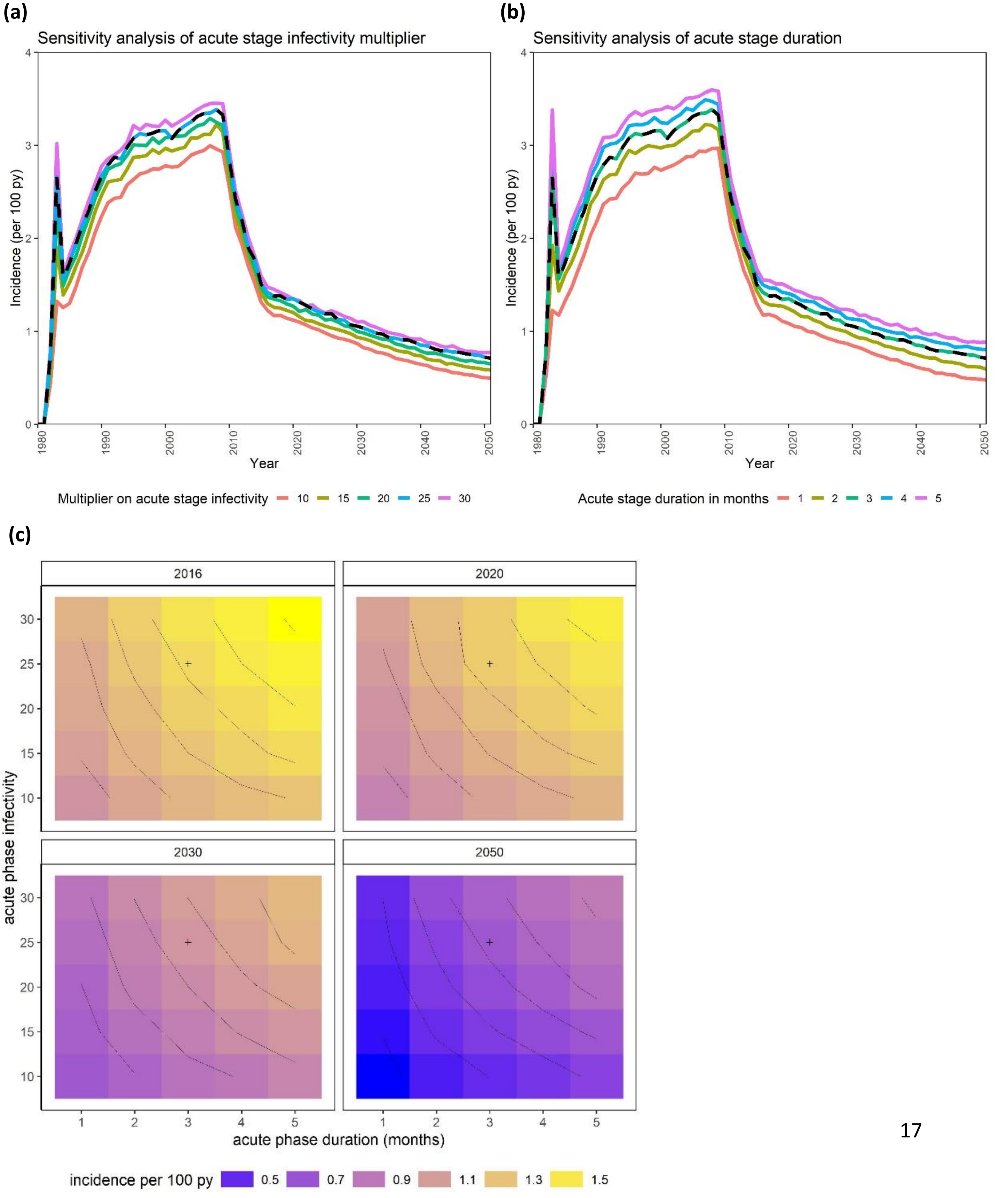

Table A3. Numerical results of the effect of varying acute stage infectivity multiplier and acute duration on incidence. Varying acute duration between 1-5 months contributed more to overall uncertainty in incidence than varying acute phase multiplier from 10-30. The combination of both parameters resulted in year-specific incidence varying by ~0.5 per 100 py (for example, in year 2050 the combination of uncertainty from both parameters resulted in incidence ranging between 0.4 and 0.9 per 100py).

	Incidence (per 100 py) range		
Year	Acute stage infectivity multiplie (acute duration set to 3 month:	Acute duration in months (acute stage multiplier set to 26)	Both parameters
2016	1.2 – 1.5	1.2 – 1.6	1.0 – 1.6
2020	1.1 – 1.4	1.1 – 1.5	1.0 – 1.5
2030	0.9 – 1.1	0.8 – 1.2	0.7 – 1.2
2050	0.5 – 0.8	0.5 – 0.9	0.4 – 0.9

2) ART efficacy and time to ART initiation

For the parameters ART_Viral_Suppression_Multiplier (ART efficacy) and Delay_Period_Mean (time from infection to ART initiation), the overall shape of the incidence curves over time also remained relatively similar to the calibrated model, and incidence was highly sensitive to ART efficacy. We tested eleven values for ART_Viral_Suppression_Multiplier (ranging from 0.8 - 1 by 0.02-unit increments), and seven values of Delay_Period_Mean (ranging from 0 - 360 days by 60-day increments). The full uncertainty analysis for each univariate and bivariate uncertainty analysis is shown below.

Figure A7. Sensitivity of HIV incidence over time in the baseline scenario (model scenario 1) varying two key parameters: a) ART efficacy, b) time from infection to ART initiation, and c) the bivariate sensitivity over each combination of the two parameter values, evaluated at four time-points (2016, 2020, 2030, and 2050). Dashed black lines in univariate and (+) in bivariate sensitivity analyses indicate actual model parameters used in final calibrated model. In each univariate sensitivity analysis, all other parameters are held according to the original calibration.

(a)

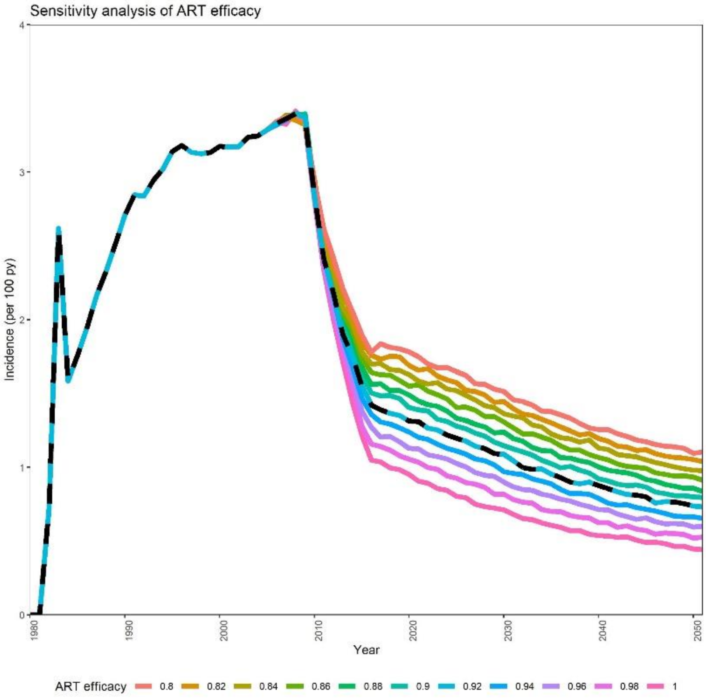

(b)

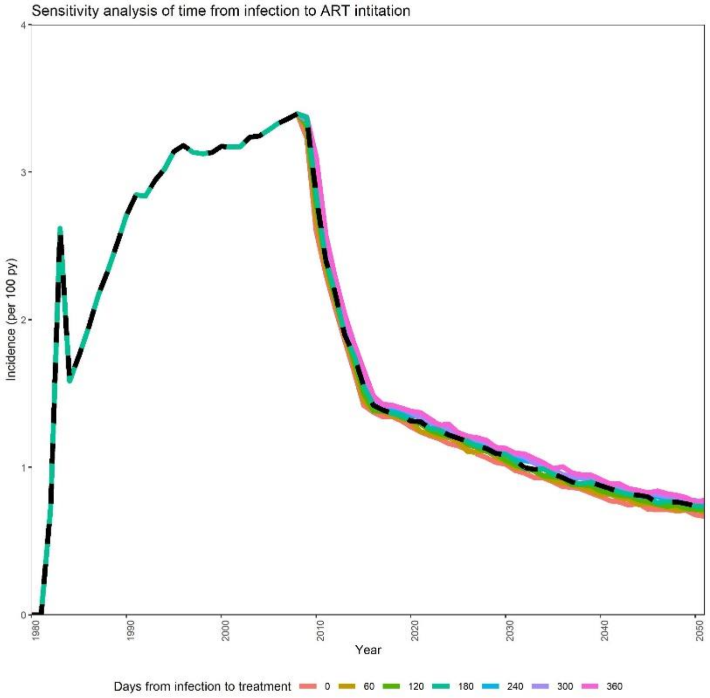

(c)

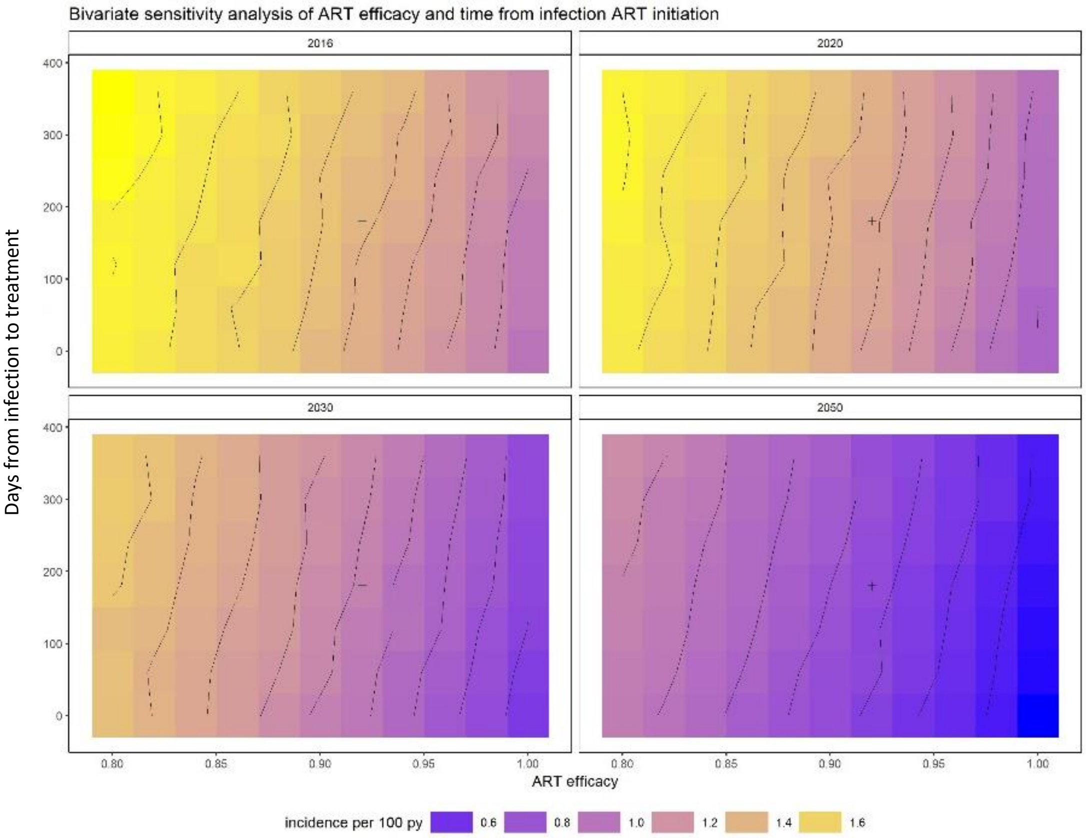

Table A4. Numerical results of the effect of varying ART_Viral_Suppression_Multiplier and the Delay_Period_Mean on incidence. Varying ART efficacy contributed substantially more to overall uncertainty than varying time from infection until ART initiation. The combination of both parameters resulted in year-specific incidence per 100 py varying from 0.8 to 0.9 per 100 py (for example, in year 2016 the combination of uncertainty from both parameters resulted in incidence ranging between 1.0 and 1.9 per 100py). Most of the variability in the results were driven by varying ART efficacy with negligible differences in incidence varying the time from infection until ART initiation.

	Incidence (per 100 py) range		
Year	ART Viral Suppression Multiplier (Delay Period Mean set to 180 days)	Delay Period Mean (ART Viral Suppression Multiplier set to 0.92)	Both parameters
2016	1.0 – 1.8	1.4 – 1.5	1.0 – 1.9
2020	1.0 – 1.8	1.3 – 1.4	0.9 – 1.8
2030	0.7 – 1.5	1.0 – 1.1	0.6 – 1.5
2050	0.4 – 1.1	0.7 – 0.8	0.4 – 1.2

3) ART efficacy and time to ART initiation

Given that our model did not predict epidemic control even under the most optimistic ART coverage, we sought to understand what intensity of test and treat could lead to theoretical epidemic control (below an incidence of 1/1000 in our model). Under the optimistic 100% ART scenario (scenario 5 in our model), three factors limit epidemic control: 1) the time from infection to diagnosis and treatment, 2) the efficacy of ART, and 3) the rate of ART dropout. We reran our model under 100% ART coverage but decreasing the time from infection to ART uptake (from 180 days to 0 days), while also varying the efficacy of ART in reducing transmission (from 92% to 100% efficacy). Epidemic control was reached only where ART efficacy was at or above 98% and time from HIV infection until ART initiation is immediate. Time from infection until ART initiation at 100% ART coverage accounted for less of the variability in HIV incidence and only had a strong effect when set to zero, or immediate uptake of ART once infected. Even initiation within an average of 2 months from infection resulted in enough onward transmission to sustain the incidence above epidemic control. This model assumes that individuals drop out of ART exponentially with a mean duration of 20 years.

Figure A8. Sensitivity of HIV incidence in the 100% ART scenario (model scenario 5) to varying two key parameters: a) ART efficacy (between 80% - 100%), b) average time in days from infection until ART initiation (between 0 and 360), and c) the bivariate sensitivity over all combinations of the two parameter estimates, evaluated at four time-points (2016, 2020, 2030, and 2050). Dashed black lines (in univariate plots) and + symbol (in bivariate plot) indicate the incidence according to actual model parameter values used in final calibrated model. In each sensitivity analysis, all other parameters are held according to the original calibration. Dashed red lines (in univariate plots) and solid red outline (in bivariate plot) indicates epidemic control threshold reached (incidence = 1/1000).

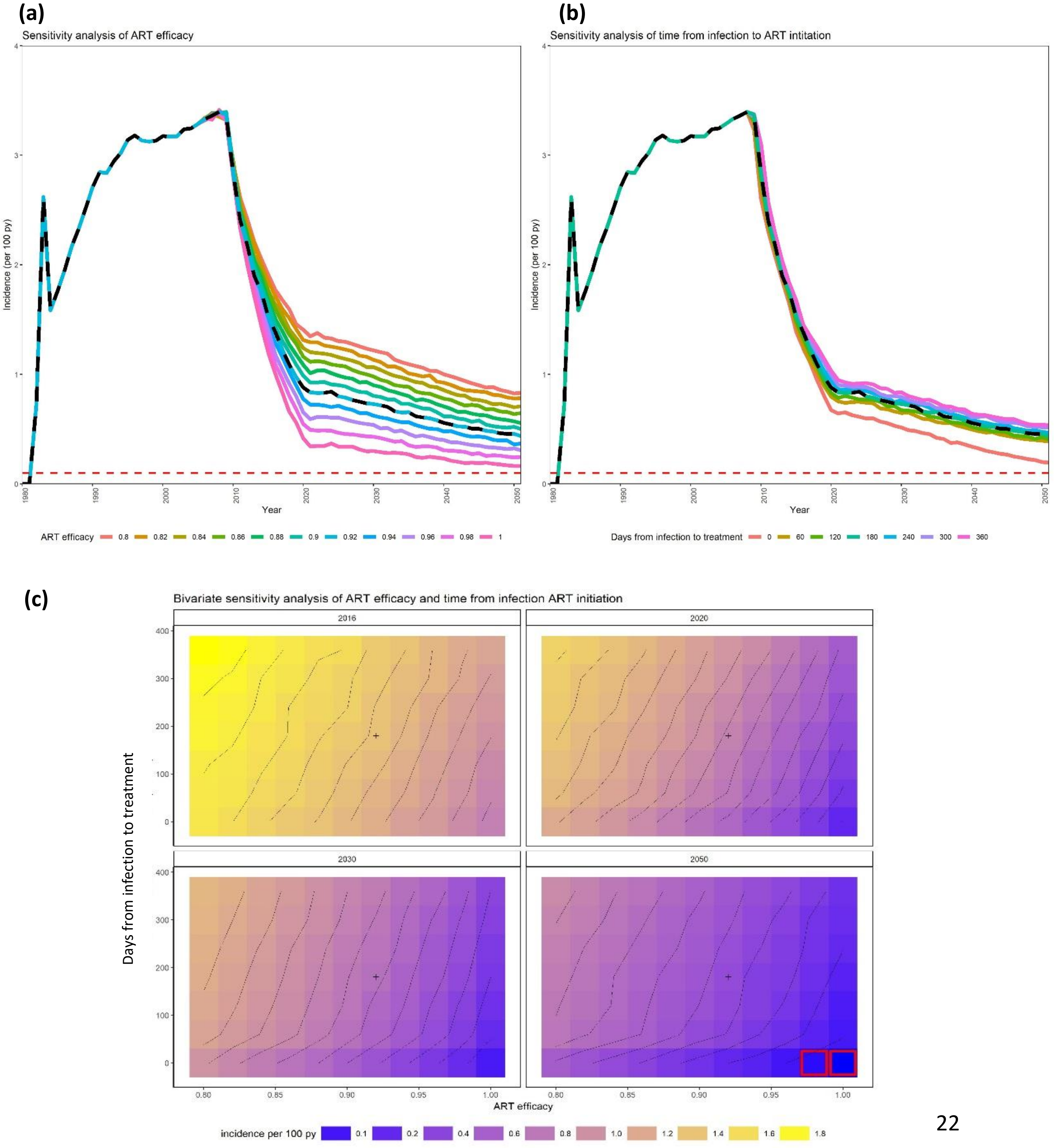

References:

1. Hollingsworth TD, Anderson RM, Fraser C. HIV-1 transmission, by stage of infection. *The Journal of infectious diseases* 2008; **198**(5): 687-93.
2. Donnell D, Baeten JM, Kiarie J, et al. Heterosexual HIV-1 transmission after initiation of antiretroviral therapy: a prospective cohort analysis. *Lancet (London, England)* 2010; **375**(9731): 2092-8.
3. Wawer MJ, Gray RH, Sewankambo NK, et al. Rates of HIV-1 Transmission per Coital Act, by Stage of HIV-1 Infection, in Rakai, Uganda. *The Journal of infectious diseases* 2005; **191**(9): 1403-9.
4. Auvert B, Taljaard D, Lagarde E, Sobngwi-Tambekou J, Sitta R, Puren A. Randomized, controlled intervention trial of male circumcision for reduction of HIV infection risk: the ANRS 1265 Trial. *PLoS Med* 2005; **2**(11): e298.
5. Bailey RC, Moses S, Parker CB, et al. Male circumcision for HIV prevention in young men in Kisumu, Kenya: a randomised controlled trial. *Lancet* 2007; **369**(9562): 643-56.
6. Gray RH, Kigozi G, Serwadda D, et al. Male circumcision for HIV prevention in men in Rakai, Uganda: a randomised trial. *Lancet* 2007; **369**(9562): 657-66.
7. Granich RM, Gilks CF, Dye C, De Cock KM, Williams BG. Universal voluntary HIV testing with immediate antiretroviral therapy as a strategy for elimination of HIV transmission: a mathematical model. *Lancet* 2009; **373**(9657): 48-57.
8. Powers KA, Ghani AC, Miller WC, et al. The role of acute and early HIV infection in the spread of HIV and implications for transmission prevention strategies in Lilongwe, Malawi: a modelling study. *Lancet* 2011; **378**(9787): 256-68.
9. Cohen MS, Dye C, Fraser C, Miller WC, Powers KA, Williams BG. HIV Treatment as Prevention: Debate and Commentary—Will Early Infection Compromise Treatment-as-Prevention Strategies? *PLOS Medicine* 2012; **9**(7): e1001232.
